# Accelerometer-Based Identification of Fatigue in the Lower Limbs during Cyclical Physical Exercise: A Systematic Review

**DOI:** 10.3390/s22083008

**Published:** 2022-04-14

**Authors:** Luca Marotta, Bouke L. Scheltinga, Robbert van Middelaar, Wichor M. Bramer, Bert-Jan F. van Beijnum, Jasper Reenalda, Jaap H. Buurke

**Affiliations:** 1 Roessingh Research and Development, 7522 AH Enschede, The Netherlands; b.scheltinga@utwente.nl (B.L.S.); j.reenalda@rrd.nl (J.R.); j.buurke@rrd.nl (J.H.B.); 2 Department of Biomedical Signals and Systems, Faculty of Electrical Engineering, Mathematics and Computer Science (EEMCS), University of Twente, 7522 NB Enschede, The Netherlands; r.p.vanmiddelaar@utwente.nl (R.v.M.); b.j.f.vanbeijnum@utwente.nl (B.-J.F.v.B.); 3 Medical Library, Erasmus University Medical Center, 3000 CA Rotterdam, The Netherlands; w.bramer@erasmusmc.nl; 4 Roessingh Rehabilitation Centre, 7522 AH Enschede, The Netherlands

**Keywords:** human movement, biomechanical phenomena, inertial measurement units, artificial intelligence, running, walking, physical activity

## Abstract

Physical exercise (PE) is beneficial for both physical and psychological health aspects. However, excessive training can lead to physical fatigue and an increased risk of lower limb injuries. In order to tailor training loads and durations to the needs and capacities of an individual, physical fatigue must be estimated. Different measurement devices and techniques (i.e., ergospirometers, electromyography, and motion capture systems) can be used to identify physical fatigue. The field of biomechanics has succeeded in capturing changes in human movement with optical systems, as well as with accelerometers or inertial measurement units (IMUs), the latter being more user-friendly and adaptable to real-world scenarios due to its wearable nature. There is, however, still a lack of consensus regarding the possibility of using biomechanical parameters measured with accelerometers to identify physical fatigue states in PE. Nowadays, the field of biomechanics is beginning to open towards the possibility of identifying fatigue state using machine learning algorithms. Here, we selected and summarized accelerometer-based articles that either (a) performed analyses of biomechanical parameters that change due to fatigue in the lower limbs or (b) performed fatigue identification based on features including biomechanical parameters. We performed a systematic literature search and analysed 39 articles on running, jumping, walking, stair climbing, and other gym exercises. Peak tibial and sacral acceleration were the most common measured variables and were found to significantly increase with fatigue (respectively, in 6/13 running articles and 2/4 jumping articles). Fatigue classification was performed with an accuracy between 78% and 96% and Pearson’s correlation with an RPE (rate of perceived exertion) between *r* = 0.79 and *r* = 0.95. We recommend future effort toward the standardization of fatigue protocols and methods across articles in order to generalize fatigue identification results and increase the use of accelerometers to quantify physical fatigue in PE.

## 1. Introduction

Physical exercise (PE) benefits human beings in many ways: from a psychological perspective, reducing anxiety and risk of depression [1]; from a physiological perspective, avoiding a sedentary lifestyle and reducing risks of cardiovascular diseases [2].; from a biomechanical perspective, reducing risk of musculoskeletal disorders (MSDs) [3]; and from a neurological perspective, improving cognitive functioning and counteracting aging processes [1]. However, PE can also lead to injuries, especially when PE activities are performed by individuals with a poor level of physical fitness [4,5,6]. Cumulative repetition of movements over time can also lead to physical fatigue, which increases the risk of MSDs instead of reducing it [7,8]. A tailored PE program for the needs of each individual would help increase fitness and avoid overloading. Timely identification of physical fatigue would be a key factor to design individualized PE programs and maximize its health benefits.

Estimation of physical fatigue in PE can be based on subjective scales (i.e., Borg’s rate of perceived exertion (RPE) [9]). However, such scales are particularly suited to capture an individual’s perception rather than the physical components of fatigue. Maximal oxygen consumption (VO2max) is considered the gold standard for fatigue estimation [10], although ergospirometers are not comfortable to wear and unsuitable for prolonged measurement sessions in daily life. Muscle fatigue is usually assessed with electromyography (EMG), considered a gold standard to measure muscle activity [11]. However, wearable EMG systems have only recently been developed, and they are yet to be validated as a tool to estimate physical fatigue. Furthermore, EMG is well-suited to capture information about acute fatigue but much less informative about cumulative fatigue generated by prolonged training sessions.

PE can also be analyzed from a biomechanical perspective. Two types of measurement systems are commonly used in modern biomechanics: optical motion capture systems (OMCs) and accelerometers. OMCs are traditionally marker-based systems that estimate an object’s position via time-of-flight triangulation (e.g., VICON) [12] and evolved recently into markerless systems. Markerless systems have the potential of being applied outside of the lab in sports and clinical applications [13] but still require expensive video cameras and have yet to be fully validated. On the other hand, accelerometers are nonintrusive, wearable, and cheap sensors that can measure accelerations in the human body. Widely used sensors that incorporate accelerometers are inertial measurement units (IMUs). They allow kinematic estimation by integrating information from an accelerometer, a gyroscope, and often a magnetometer (IMMUs), for example, using a Kalman filter to estimate individual sensor orientation and combining IMU outputs with a biomechanical model [14]. They have the possibility to continuously measure data in a wide variety of real-life applications, ranging from work tasks [15,16,17] to PE [18,19] and assessment of injury risk in running [20,21].

A promising approach to overcome the use of subjective scales and avoid intrusive measurement techniques (i.e., EMG and ergospirometry) is to study physical fatigue using a biomechanical approach, in particular, identifying changes that happen in movement patterns over time. In fact, lower limb changes in kinetic, kinematic, and spatiotemporal parameters due to fatigue have been found in a wide range of activities. The scientific literature has focused particularly on work activities, where fatigue has been assessed by means of changes in heart rate, tremor, gait parameters, and coordination between segments in the lower limbs and trunk [15]. In PE, running has been mostly investigated. Changes in lower limbs joint angles [22,23,24] and segmental accelerations [25,26,27] have been found in running activities ranging from short recreational runs to marathons. The increase in data availability and computer power in recent years has paved the way for the use of machine learning in biomechanical research. Machine learning is a subfield of artificial intelligence that aims to identify, estimate, or classify outcomes (e.g., fatigue) by learning from examples [28]. Accelerometer-derived data are particularly suited for machine learning algorithms, which have the advantage of learning from large amounts of data to predict outcomes. Still, the types of problems that are better addressed by machine learning than traditional biomechanics are yet to be established [29].

While a large amount of the literature is present regarding the ability of wearables to identify, estimate, and monitor fatigue in the workplace [30], the main focus in the field of accelerometers and PE or sports activities concerns performance and quality of movement rather than fatigue [31,32,33]. A comprehensive systematic review regarding accelerometer-based identification of physical fatigue in the lower limbs during PE is still lacking, as well as an interpretation and comparison of results obtained with traditional biomechanical and machine learning approaches.

In this study, we aim to contribute to state-of-the-art PE monitoring with a comprehensive overview of the performance of accelerometer-based methods to identify fatigue in cyclical PE, since cyclical tasks allow for a comparison across different PE activities. A literature search is performed including articles that assess biomechanical changes in fatiguing cyclical PE activities or use such changes to identify a fatigued state. We aim to provide an overview of the literature regarding accelerometer-based measures of biomechanical changes due to fatigue, as well as an overview of the literature regarding the detection of fatigue via models or machine learning approaches that use kinematic features measured via accelerometers.

## 2. Materials and Methods

### 2.1. Search Strategy

This review was reported following the PRISMA guidelines (Table S1, Supplementary Materials) [34]. An exhaustive search strategy was developed by an experienced information specialist (WMB). The search was developed in Embase.com, optimized for sensitivity, and then translated to other databases following the method described by Bramer et al. [35]. The search was carried out in the following databases: Embase.com (date of inception 1971), Medline ALL via Ovid (1946 to Daily Update), Web of Science Core Collection, and CINAHL Plus via EBSCOhost. After the original search was performed on 5 August 2020, the search was last updated on 31 May 2021 using the methods described by Bramer et al. [36].

The search strategies for Embase and Medline used relevant thesaurus terms from Emtree and Medical Subject Headings (MeSH), respectively. In all databases, terms were searched in the titles and abstracts of references. The search contained terms for: (1) fatigue or exhaustion; (2) physical exercise, gait, walking, or running; and (3) inertial measurements or accelerometry. Terms were combined with the Boolean operators AND and OR, and proximity operators were used to combine terms into phrases. The full list of the keywords used in each search strategy for all four databases is available in Appendix A (Table A1). The searches in Embase and Web of Science were limited to exclude conference papers older than 3 years. In all databases, non-English articles and animal-only articles were excluded from the search results. No study registries were searched. The reference lists of retrieved nonincluded relevant review articles and of the included references, as well as articles citing these papers, were scanned for relevant references missed by the search. No authors or subject experts were contacted, and we did not browse unindexed journals in the field. The references were imported into EndNote, and duplicates were removed by the medical librarian (WMB) using the method described by Bramer et al. [37].

### 2.2. Screening of Articles and Eligibility Criteria

Two reviewers (LM and BS) independently screened titles and abstracts in EndNote using the method described by Bramer et al. [38]. Any discrepancies in the verdict were resolved by discussion with a third reviewer (JR). A total of seven exclusion criteria in the abstract screening phase (Table A2) and eight exclusion criteria in the full-text screening phase (Table A3) were used and can be found in Appendix A. Two reviewers (LM and BS for the first half of the articles in alphabetical order, and LM and RvM for the second half of articles) independently screened the full-text articles. Any discrepancies in the verdict were resolved by discussion with a third reviewer (JR).

The aim of this review was to assess accelerometer-based methods to identify fatigue in cyclical PE. In the initial search strategy, work activities were still included. However, the recovery time and intensity of such activities have very large variations compared to cyclical individual PE activities (i.e., running, walking, jumping, and stair climbing). Furthermore, work task movement patterns lack continuity when compared to cyclical PE tasks and were, therefore, excluded (EC2.2, Table A3).

### 2.3. Data Extraction

**Figure 1:**
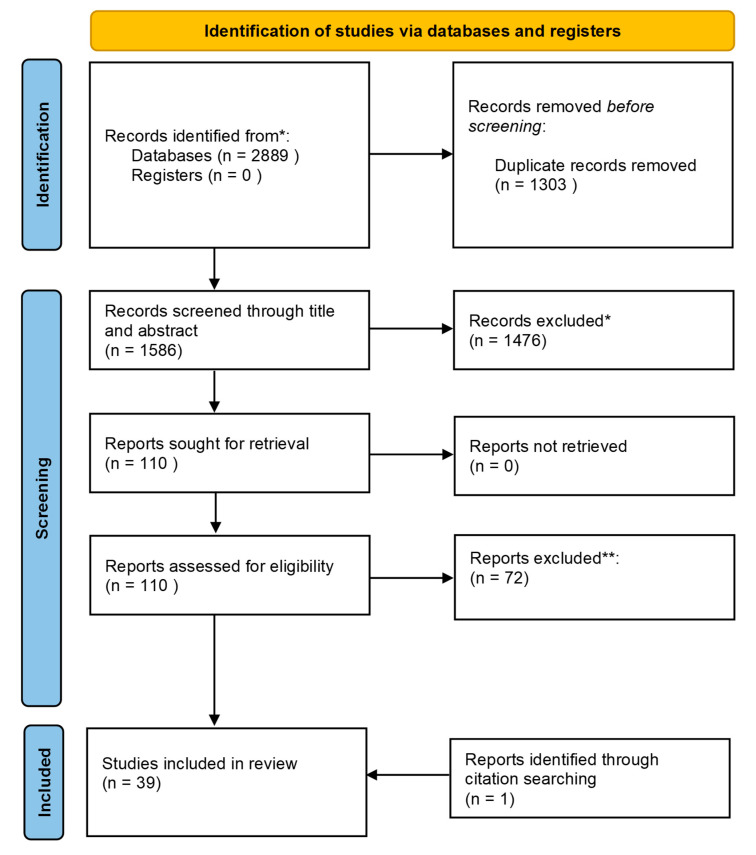
Flow of systematic review process according to PRISMA diagram after first search. * Records excluded via exclusion criteria in Table A2. ** Records excluded via exclusion criteria in Table A3.

A total of 2889 articles were retrieved, resulting in a total number of 39 articles included in this review after the screening process (Figure 1). After the initial search (5 August 2020), removal of duplicates, screening of titles, and abstract and screening of full-texts, thirty articles were included (Supplementary Material, Figure S1). After performing the search a second time (31 May 2021), eight new articles were identified (Supplementary Material, Figure S1). One article was identified through citation searching.

### 2.4. Outcomes of Interest

All articles included in this review aimed to identify fatigue in the lower limbs during PE using accelerometers. Articles that aimed to quantitatively identify changes due to fatigue in lower limb biomechanics were classified as Type I. The outcomes of interest for these articles were biomechanical parameters measured before and after a fatiguing intervention. Biomechanical parameters were kinematic or spatiotemporal variables that can be measured directly using accelerometers: segment accelerations, shock attenuation, and stride parameters. Articles that aimed to identify, predict, or classify fatigue states based on quantitative biomechanical features were classified as Type II. The outcomes of interest for Type II articles were the performance metrics of the proposed model or classifier. Additional variables for which data were sought concerned study protocol, subject population, measurement system, intervention (fatigue protocol), fatigue reference, and data analysis techniques.

#### 2.4.1. Segment Accelerations

The lower limb accelerations considered in this review were feet, tibia, thigh, and sacrum segmental accelerations. Peak segmental accelerations are commonly used to understand human motion, and they have been linked, in particular, to tibial bone loading [39], which could provide relevant information in understanding injury risk. Peak tibial accelerations are commonly used as an indirect measure of impact during running [27].

#### 2.4.2. Shock Attenuation

Shock attenuation is the magnitude or frequency reduction of the impact shock wave in human movements that involve an impact of the lower limbs with the ground [40]. Shock attenuation strategies are used by the body to deal with high impacts with the ground that can happen during various PE activities [27]. The shock attenuation outcomes considered in this review were between the tibia and sacrum, trunk, or forehead.

#### 2.4.3. Stride Parameters

Stride spatiotemporal parameters are commonly used to describe the human gait (e.g., stride frequency, stride length, and stride time). Stride parameters can be related to cumulative load and contain relevant information to prevent running-related injuries [41], and were, therefore, considered in this review.

#### 2.4.4. Model Performance Metrics

For Type II articles, models were built to identify or classify fatigue states. The performances of these models were evaluated by means of accuracy metrics, typically used in classification problems, or correlation metrics, typically used in regression problems. The accuracy, sensitivity, and specificity of the classifier are common performance metrics in classification problems, while root mean squared error (RMSE) and Pearson’s r are frequently used correlation metrics.

### 2.5. Quality Assessment

A quality assessment checklist was adapted from the Downs and Black checklist [42], tailoring criteria regarding reporting, internal and external validity, and power. Twelve items were selected and used for Type I articles. Additionally, items adapted from the Luo et al. *Guidelines for Developing and Reporting Machine Learning Predictive Models in Biomedical Research* [43] were added for the quality assessment of Type II articles. Four items were selected and used to replace Downs and Black items in order to tailor the checklist to Type II articles. The full quality assessments for Type I (Table S2) and Type II (Table S3) articles are reported in the Supplementary Materials. Selected items from the two quality assessment checklists are shown in Table 1. The maximum score for both types of articles was 11; articles that did not exceed a threshold of 5/11 were discarded.

## 3. Results

### 3.1. Overview of Article Characteristics

Articles that passed the screening phase and were included in the review are shown in Table 2. The article identification process led to 39 articles. PE activities included running (28 articles), walking (4 articles), jumping exercises (4 articles), stair-climbing tests (SCTs) (2 articles), and gym exercises (1 article). The aim of this section is to summarize the subject populations, sensor placements, fatiguing protocols, fatigue references, and outcomes of interest for the included articles.

#### 3.1.1. Subject Population

The subject population characteristics are shown in Table 2. The number of subjects varied from 3 to 222 (running, 3–222; walking, 9–24; jumping, 8–30; SCTs, 20–21; gym exercises, 14). The average number of participants across all articles was 23.1 ± 33.8 (mean ± SD). A total of 18 articles reported quantitative information on the training levels of their participants. In running, the reported pieces of information were training load (33.2 ± 13.5 km/week across nine articles) and frequency (2.4 ± 0.89 times per week across five articles). In all other PE activities, the reported information regarded the time spent per week being physically active or exercising (0.5 h/day in one jumping article, two times per week in two walking articles, and 4.5 h/week in one gym exercising article).

**Table 2:** Subject population and measurement system characteristics.

		Subject Population	Measurement System
**Authors [ref]**	**Cyclical PE Activity**	**Type and N° of Subjects** **Sex** **Age (years)** **Height (cm)** **Mass (kg)** **Subject Eligibility and Training Level**	**Accelerometer(s) Characteristics and n° of Axes** **N° of Accelerometers and Placement** **Weight** **Operating Range** **Sampling Frequency** **Manufacturer (Model)**
Abt et al. [44]	Running	12 competitive runners M and F 24.5 ± 4.1 174 ± 9 65.2 ± 9.8 Running for at least 3 yrs; min. of 48 km/wk; min. pace of 10.7 km/h; no injuries in the last 3 months	3D accelerometer 2: tibia and forehead 21 g ±25 G 1200 Hz Silicon Designs, Inc., Issaquah, WA, USA (Model 2422-010)
Ameli et al. [19]	Stair climbing	20 subjects 10 M; 10 F M: 27.3 ± 2.3; F: 24.4 ± 3.7 / BMI (kg/m^2^) M: 22.5 ± 2.8; F: 21.6 ± 2.8 Not overweight; age 20–30; no stationary occupation; no sign of pain during motion	3D IMU 9: left foot, right foot, left tibia, right tibia, left thigh, right thigh, sacrum, sternum, head / / 60 Hz Xsens Technologies BV, Enschede, The Netherlands
Arias-Torres et al. [45]	Walking	9 subjects Sex NS 21.2 ± 3.2 167 ± 7 67.5 ± 11.5 Subject eligibility NS	3D accelerometer (embedded in smartphone) 1: sacrum / / 100 Hz Bosch Sensortech BMA220
Bergmann et al. [46]	Stair climbing	21 subjects 16 M; 5F 32 (range 23–58) 177 ± 8 75 ± 9 Subject eligibility NS	3D IMU 7: left foot, right foot, left tibia, right tibia, left thigh, right thigh, sacrum / / 100 Hz Xsens Technologies BV, Enschede, The Netherlands (Model MTx)
Brahms et al. [47]	Running	16 elite (E) distance runners + 16 recreational (R) runners Sex NS E 21.2 ± 3.0; R 26.8 ± 4.8 E 175.6 ± 8.7; R 174.5 ± 7.4 E 63.4 ± 9.5; R 71.6 ± 10.5 E: trained 4 days/wk in prev 2 years; competed within preceding year R: trained up to 3 h/wk Both: no history of lower extremity surgery; no running-related injury in previous year	3D IMU 1: right foot / 160 m/s^2^ 100 Hz Xsens Technologies BV, Enschede, The Netherlands (Model MTw)
Butler et al. [48]	Running	12 high arch (HA) + 12 low arch (LA) recreational runners Sex NS HA 20.9 ± 3.0; LA 21.8 ± 3.2 HA 170 ± 7; LA 173 ± 11 HA 68.4 ± 5.8; LA 70.0 ± 7.3 Running min of 10 miles/wk; age between 18–40; no lower extremity or cardiovascular conditions	1D accelerometer 1: tibia 2.83 g (including aluminum case) / 1080 Hz PCB Piezotronics Inc., Depew, NY, USA
Clansey et al. [23]	Running	21 distance runners M 36.2 ± 12.5 180 ± 8 75.4 ± 11.5 No musculoskeletal injury; training average of 72 ± 34 km/wk	2D accelerometer 2: forehead and distal tibia / 16 g Noraxon, Scottsdale, AZ, USA
Clermont et al. [49]	Running	27 runners 12 M; 15 F M: 50.4 ± 13.0; F: 40.9 ± 10.3 M: 174.9 ± 10.3; F: 160.5 ± 4.3 M:79.0 ± 12.0; F: 58.2 ± 7.8 Registered officially for a marathon race; age > 18; no lower extremity or cardiovascular conditions, no use of foot orthoses	3D IMU 1: sacrum / / 100 Hz Lumo Bodytech Inc., Mountain View, CA, USA (Lumo Run)
Coventry et al. [50]	Drop jumping	8 subjects M 23.8 ± 2.4 184 ± 7 81.6 ± 6.8 No history of lower extremity injuries in prev. 6 months; physically active approx. 30 min/day	1D accelerometer 2: distal anteromedial tibia and forehead 1.7 g / 1000 Hz PCB Piezotronics Inc., Depew, NY, USA (Model 353B17)
Derrick et al. [51]	Running	10 recreational runners Sex NS 25.8 ± 7.0 Height NS 70.8 ± 10.1 Injury-free and physically active	1D accelerometer 2: distal anteromedial right tibia and forehead 1.8 g / 1000 Hz PCB Piezotronics Inc., Depew, NY, USA (Model 353B17)
Encarnacion-Martinez et al. [52]	Running	17 recreational runners M 28.7 ± 8.3 178 ± 7 72.2 ± 8.2 Running min of 2/wk and more than 20 km/wk in prev year; no injuries in prev 6 months	3D accelerometer 2: distal anteromedial tibia (DL) and forehead 2.5 g ±16 G 300 Hz Blautic, Valencia, Spain
Garcia Perez et al. [53]	Running	20 recreational runners 11 M; 9 F 34 ± 8 172 ± 8 63.6 ± 8.0 Subject eligibility NS, training for 4.2 ± 1.0 days/wk and 49.8 ± 17.8 km/wk	1D accelerometer 2: proximal anteromedial right tibia and forehead 55 g / Freescale Semiconductor, Munich, Germany (MMA7261QT)
Hajifar et al. [17]	Walking	24 subjects (Lab study 2) 12 M; 12 F 22.7 ± 3.9 170.3 ± 11.1 68.3 ± 11.7 No recent history of MSD or lower body injury; exercising 2–3 days/wk	3D IMU (embedded in smartphone) 1: tibia / ±160 m/s^2^ 100 Hz InvenSense Inc., San Jose, CA, USA (MPU-6500)
Hardin et al. [54]	Running	24 recreational runners (8 soft midsole (SM), 8 medium midsole (MM), 8 hard midsole (HM)) M Age NS SM: 176 ± 3; MM: 172 ± 4; HM: 177 ± 5 SM: 71.6 ± 6.8; MM: 68.4 ± 8.2; HM: 75.5 ± 7.0 No lower extremity injury; previous treadmill-running experience	1D accelerometer 1: distal anteromedial right tibia 1.7 g (3.8 g considering aluminum bracket) / 1000 Hz PCB Piezotronics Inc., Depew, NY, USA (Model 353B17)
Hoenig et al. [55]	Running	30 runners (15 recreational (R) 15 competitive (C)) R: 25.3 ± 7.6; C: 28.7 ± 4.3 \ BMI (kg/m^2^) R: 23.7 ± 2.8; C: 22.4 ± 1.8 No injury or pain impairing movement in prev 3 months; no history of gait disorder; no use of insoles to correct orthopedic disorders	3D IMU 3: right foot, sacrum, sternum / / 100 Hz Xsens Technologies BV, Enschede, The Netherlands (Model MTw)
Jiang et al. [56]	Gym exercises	14 subjects 12 M; 2F 27.4 ± 4.2 176 ± 7 74.1 ± 12.1 Subject eligibility NS; exercising 4.5 ± 3.4 h/wk	3D IMU 9: left foot, right foot, left tibia, right tibia, left thigh, right thig, sacrum, sternum, head / / 240 Hz Xsens Technologies BV, Enschede, The Netherlands
Karvekar et al. [57]	Walking	24 subjects (Lab Study 2) 12 M; 12 F 22.7 ± 3.9 170.3 ± 11.1 68.3 ± 11.7 No recent history of MSD or lower body injury; exercising 2–3 days/wk	3D IMU (embedded in smartphone) 1: tibia / ±160 m/s^2^ 100 Hz InvenSense Inc., San Jose, CA, USA (MPU-6500)
Lucas Cuevas et al. [58]	Running	38 recreational runners 20 M; 20 F 29.8 ± 5.3 170.3 ± 11.4 65.4 ± 10.1 No injuries in prev year; no surgery in prev 3 years; no prev use of insoles; training routine min of 20 km/wk	3D accelerometer 2: proximal anteromedial tibia and forehead 2.5 g / 500 Hz Sportmetrics, Spain
McGinnis et al. [59]	Vertical jumping	21 subjects 15 M; 6 F M: 19.7 ± 1.1; F: 20.2 ± 1.0 M: 178.7 ± 6.9; F: 172.4 ± 4.9 M:78.0 ± 9.6; F: 68.0 ± 8.1 Experience exercising under fatigue condition similar to the study protocol	3D IMU 1: sacrum / / 300 Hz Yost Engineering, Portsmouth, OH, USA (YEI 3-Space)
Meardon et al. [60]	Running	9 recreational runners Sex NS 25.9 ± 8.5 170.2 ± 10.9 62.6 ± 8.3 No history of overuse injury; training volume of 30.3 ± 9.7 km/wk	1D accelerometer 1: distal anteromedial tibia / ±50 G 1000 Hz Analog Devices, Wilmington, Massachusetts, USA (ADXL250)
Mercer et al. [61]	Running	10 recreational runners M 24 ± 6 184 ± 10 78.4 ± 9.6 Physically active; injury-free; experienced running on treadmill	1D accelerometer 2: right tibia and forehead 6.7 g ±50 G 1000 Hz Kistler, Amherst, NY, USA (8628B50)
Meyer et al. [62]	Running	12 recreational runners 8 M; 4 F 36 ± 10 178 ± 7 72 ± 6 Older than 18; training min of 2 times per week; no running-related injury in prev 6 months	3D IMU 2: left and right foot / ±16 G 512 Hz Physilog 5, Gait Up, SA, CH
Mizrahi_a_ [63], Mizrahi_b_ [64], Mizrahi_c_ [65]_,_ Mizrahi_d_ [66] et al.	Running	14 recreational runners M 24.2 ± 3.7 175.5 ± 5.9 73.2 ± 8.3 No history of injury	1D accelerometer 2: proximal tibia and sacrum 4.2 g / 1667 Hz Kistler PiezoBeam, Kistler, Switzerland (8634B50)
Moran et al. [18]	Drop jumping	15 physically active subjects M 21.4 ± 1.5 178 ± 15 80.1 ± 5.84 No history of lower extremity injury; competency requirements in drop-jumping: no horizontal travel between take-off and landing, no excessive pause between loading and propulsion, short duration landing phase, and toe-first landing pattern	1D accelerometer 1: proximal right tibia 17 g ±50 G 1000 Hz Analog Devices, Ireland (ADXL250)
Morio et al. [67]	Running	8 recreational runners M 26 ± 2 178 ± 6 74 ± 11 Free of lower limb injury	3D accelerometer 1: distal tibia / ±50 G 100 Hz Endevco, Depew, NY, USA (Isotron)
Provot_a_ [68], Provot_b_ [69] et al.	Running	10 recreational runners 5 M; 5 F 38.0 ± 11.6 173 ± 10 66.3 ± 12.6 Training frequency of 2 sessions/wk, recent competition record for 10 km (<45 min) or half-marathon (<100 min)	3D IMU 3: right foot, medial right tibia, sacrum 22 g ±24 G (foot and tibia) ±8 G (sacrum) 1344 Hz IMU Hikob Fox, Villeurbanne, France
Reenalda_a_ et al. [22]	Running	3 experienced runners M 38.7 ± 8.2 182 ± 2.4 73 ± 3.7 No injuries prev year; expected marathon finish time of 3 h	3D IMU 8: left foot, right foot, left medial tibia, right medial tibia, left upper leg, right upper leg, sacrum, sternum 27 g ±160 m/s^2^ 60 Hz Xsens Technologies BV, Enschede, The Netherlands (Model MTw)
Reenalda_b_ et al. [27]	Running	10 experienced runners M 31 ± 5 183 ± 3 76 ± 9 No injuries prev 6 months; training load min of 40 km/wk	3D IMU 8: left foot, right foot, left medial tibia, right medial tibia, left upper leg, right upper leg, sacrum, sternum 30 g ±18 G 100 Hz Xsens Technologies BV, Enschede, The Netherlands (Model MTx)
Ruder et al. [26]	Running	222 marathon runners 119 M; 103 F 44.1 ± 10.8 / / Not injured; age > 18 years	3D accelerometer 1: distal tibia 12 g ±16 G 1000 Hz IMeasureU BlueThunder, Auckland, New Zealand
Sandrey et al. [70]	Vertical jumping	30 active subjects 15 M; 15 F 21.5 ± 5.04 173.5 ± 12.7 72.65 ± 16.4 No history of lower extremity injury	3D accelerometer 1: proximal tibia / ±50 G 1000 Hz BIOPAC Systems Inc., Goleta, United States (TSD109C)
Schutte_a_ et al. [71]	Running	20 runners 12 M; 8 F 21.05 ± 2.14 177 ± 8 66.12 ± 6.19 No injuries in prev 6 months; training volume of 48.28 ± 36.18 km/wk	3D accelerometer 1: sacrum 48 g ±16 G 400 Hz Gulf Coast Data Concepts, MS, USA (X16-2)
Schutte_b_ et al. [25]	Running	16 recreational runners 10 M; 6 F 20.13 ± 0.72 174.75 ± 7.34 63.06 ± 9.45 No history of injuries, training load of 26.44 ± 6.26 km/wk	3D accelerometer 2: distal right tibia and sacrum 33 g ±50 G 1024 Hz Gulf Coast Data Concepts, MS, USA (X50-2)
Strohrmann et al. [72]	Running	21 runners (different skills levels) / / / / Training loads: beginners 0–5 km/wk; intermediate 5–25 km/wk; advanced 25–45 km/wk; expert > 45 km/wk	3D IMU 1: sacrum 22 g ±6 G 100 Hz ETHOS
Verbitsky et al. [73]	Running	22 subjects M 30.8 ± 5.1 173.9 ± 7.3 70.4 ± 9.2 Training min of 2 times per week; no history of injury	1D accelerometer 1: tibia 2.3 g / 1667 Hz PCB (A303)
Zhang et al. [74]	Walking	17 subjects 9 M; 8 F 29 ± 11 174 ± 10 73 ± 12 Non-sedentary, independent and free of MSDs; no use of medication; no balance or vision disorders	3D IMU 2: right tibia and sternum / / 120 Hz MMA7261QT

M: male, F: female, E: elite, C: competitive, R: recreational, NS: not specified, HA: high arch, LA: low arch, SM: soft midsole, MM: medium midsole, HM: hard midsole, DL: dominant leg, BMI: body mass index, MSD: musculoskeletal disorder.

#### 3.1.2. Measurement System and Sensor Placement

The measurement systems used to identify biomechanical parameters were simple accelerometers (20 articles) or accelerometers embedded in IMUs (19 articles). In three cases, the accelerometers or IMUs were embedded in a smartphone.

The accelerometers were fixed to a single body segment in 16 articles (10 tibia, 5 sacrum only, and 1 foot) and to multiple body segments in 23 articles. In particular, the tibia was chosen as a sensor location in 78% of the running articles and 79% of all articles, while the percentage of the sacrum placement was consistent at 46%. The foot and thigh were chosen as sensor locations for only 26% and 13%, respectively, out of all articles. A summary of the sensor placement for all articles and each activity can be found in Table 3.

The accelerometers were attached on both limbs in only six articles. Placement of the accelerometer was reported also for the forehead (10 articles) and sternum (6 articles), since they are needed for computing shock attenuation. Measurement systems characteristics and placement are reported in detail in Table 2.

#### 3.1.3. Fatiguing Protocol

**Figure 2:**
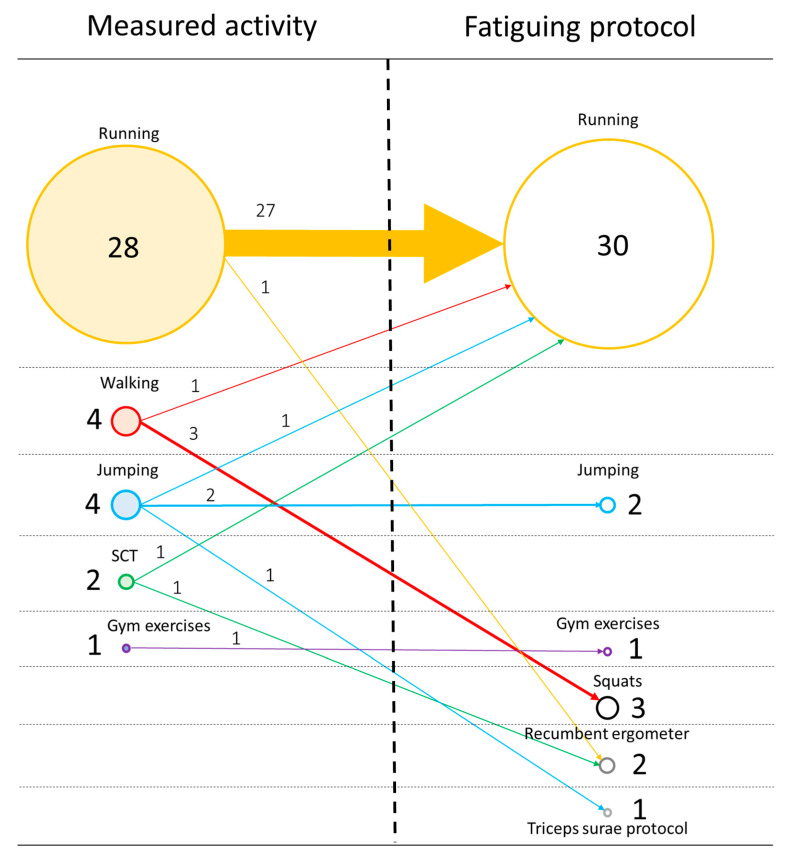
Summary of fatiguing protocols and measured activities. Circles on the left side represent the number of articles that measured each PE activity, while circles on the right side represent the number of articles for each PE activity chosen as a fatiguing protocol. Horizontal arrows represent the articles that used the same fatiguing protocol PE activity as the measured activity, while diagonal arrows represent the articles that chose a different PE activity.

Fatiguing protocols varied across the articles. A comprehensive summary of the measured activities and related fatiguing protocols across all the articles can be found in Figure 2. A total of 30/39 articles (77%) reported a fatiguing protocol consisting of the same activity as the main measured activity. All six articles that reported walking and SCT as the main measured activity used a different activity as a fatiguing protocol.

All articles reported the intensity of the fatiguing protocol by means of duration, length, speed, or number of repetitions for each PE activity. For running activities, the average reported duration of the fatiguing protocol was 28.8 min (range of 13.6–48.5 min) with a speed of 3.61 m/s (range of 2.75–4.39 m/s), while the average reported distance was 9.5 km (range of 3.2–35.0 km). Two running protocols lasted only between 5 and 10 min but consisted of running on a graded treadmill with a slope of 3–7.5%. The squat frequency consisted of 8–22 repetitions per minute. Gym exercises varied between 3 and 52 sets of 5 repetitions of squats, high knee jacks, and toe touches. The sledge ergometer durations were between 9 and 10 min. The frequency of the triceps surae protocol was 23 heel raises per minute. The fatiguing protocol characteristics are reported in detail for all articles in Table 4.

The stopping criteria of the fatiguing protocols varied per PE activity. Out of 30 articles that reported a running fatiguing protocol, nine articles based the stopping criteria on the length of the run; nine articles based it on a threshold for RPE, HR, or end-tidal carbon dioxide pressure (PETCO2); five articles let participants run until exhaustion; and three articles based their stopping criteria on a decrease in performance, while four articles did not report clear stopping criteria. A decrease in performance was also used as stopping criterium for the two articles reporting a jumping fatiguing protocol and three articles reporting squatting, recumbent ergometer, and triceps surae fatiguing protocols. The only protocol based on gym exercises used a stopping criterion of subject exhaustion, while the remaining two articles reporting squatting fatiguing protocols used an RPE threshold as the stopping criterion. Finally, one recumbent ergometer fatiguing protocol was reported to be stopped when subjects felt uncomfortable.

#### 3.1.4. Fatigue Reference

The fatigue reference metrics across all the articles are reported in Table 5. A total of 19 out of 39 articles (49%) reported RPE as a fatigue reference. The RPEs consisted solely of Borg’s RPE [9] (either on a 6–20 or 1–10 scale) for all fatiguing protocols, except for the recumbent ergometer, which also included perceived muscle soreness as a fatigue reference. The HR parameters consisted of changes in HR, absolute HR values, and relative changes compared to the HR max and accounted for 15% of all articles. The ventilatory parameters consisted of changes in PETCO2 and VO2 max, accounting for 18% of articles. Other physiological parameters included changes in creatine kinase and blood lactate concentration. A total of 5 out of 39 articles combined multiple fatigue references in their protocols.

#### 3.1.5. Outcomes of Interest

A total of 32 articles evaluated changes due to fatigue in lower limb biomechanics (Type I), and 7 articles used machine learning approaches to identify, classify, or predict fatigue stages (Type II). Performance metrics were chosen by all seven Type II articles and are presented in Section 3.4.

For Type I articles, peak tibial acceleration (PTA) was the most common reported outcome, chosen in 13 running articles and 3 jumping articles. Shock attenuation was reported in 11 articles: seven times between head and tibia (six running, one jumping) and two times between sacrum and tibia (running). Peak sacral acceleration (PSA) was reported in five articles (four running, one jumping) and peak foot acceleration (PFA) in one running article. Other acceleration-based variables that were reported in running articles were tibial acceleration reduction, tibial impact rate, and peak-to-peak tibial acceleration.

Stride spatiotemporal parameters were chosen both in running and jumping articles. Stride length was the most common variable (six articles), followed by step frequency, stride frequency, stride time, and contact time (each reported in two articles). Step length and foot strike angle (FSA) were also reported in one article each.

Other variables were also chosen in different activities. In running, some articles focused on frequency domain parameters (i.e., local dynamic stability, power spectral density, and signal power magnitude). Center of mass (COM) displacement was chosen in one running article, while one jumping article reported vertical displacement of the sacrum. Other reported outcomes in jumping articles were touchdown angle, peak tibial angular velocity, and maximal vertical velocity and acceleration of the sacrum. In SCTs, the ranges of motion of the ankle, knee, thigh, and trunk were reported.

### 3.2. Quality Assessment

Quality assessment scores for each article are shown in Table 4. All 39 articles that were evaluated after full-text screening exceeded the threshold of 5/11. The overall quality assessment score was 9.3 ± 1.3 (9.2 ± 1.3 for Type I and 9.6 ± 1.1 for Type II articles). A complete assessment of all the quality assessment items for each article can be found in the Supplementary Materials (Tables S2 and S3).

### 3.3. Overview of Biomechanical Changes Due to Fatigue

#### 3.3.1. Running

The changes due to fatigue in biomechanical parameters for running activities can be found in Table 6. Increasing PTA with fatigue was found in 11/13 articles. In 2/13 articles, PTA increased or decreased with fatigue depending on different conditions (i.e., running environment and shoe characteristics). A total of 6/13 articles found significant increases of PTA with fatigue, while 1/13 articles found a significant decrease of PTA. PSA was found to increase with fatigue in 4/4 articles, although only 2/4 articles found the increase to be significant. PFA was found to increase with fatigue by 1/1 article, although the increase was significant only for recreational runners. Shock attenuation between the head and tibia increased in 3/6 articles, while in 2/6 articles, it was found to increase depending on different conditions (i.e., shoe characteristics) or mathematical calculations (transfer function vs. ratio). A total of 2/6 articles found a significant increase in head-to-tibia shock attenuation with fatigue, while 1/6 articles found a significant decrease. Furthermore, 2/2 articles found an increase in sacrum-to-tibia shock attenuation with fatigue, one of them being significant. Significant changes in stride and step spatiotemporal parameters were found in 5/16 articles (1/6 found significant increase in stride length; 1/2 in stride frequency; 0/2 in stride time; 1/2 in step frequency; 1/2 in contact time; 0/1 in step length; 1/1 found significant decrease in FSA).

Significant changes in fatigue reference between the fatigued and non-fatigued states were found in all five articles that reported them. A significant increase in RPE with fatigue was found in one article; a significant increase in oxygen consumption was found in one article; a significant increase in heart rate was found in one article; and a significant decrease of end-tidal carbon dioxide pressure was found in two articles. The average RPE in the fatigued state was reported by four articles and was equal to 15.7 ± 1.4 (6–20 Borg Scale).

#### 3.3.2. Walking

All four walking articles were categorized as Type II articles and are reported in Section 3.4.

#### 3.3.3. Stair-Climbing Test

A total of 1/2 SCT articles investigated changes in biomechanical parameters. An increase in ankle, knee, thigh, and trunk range of motion (ROM) was found with fatigue during descent, the trunk ROM being the only one showing a significant difference. Non-significant increases were found with fatigue in knee, thigh, and trunk ROM during ascent, while a non-significant decrease with fatigue was found in ankle ROM.

#### 3.3.4. Jumping Exercises

Changes due to fatigue in biomechanical parameters for jumping activities can be found in Table 7. A total of 3/3 jumping articles found an increase in PTA with fatigue. A significant increase in PTA with fatigue was found in one article only in shorter jumps (30 cm), while it was non-significant in higher jumps (50 cm). Another article found a significant increase in PTA with fatigue in landing, but a non-significant increase during take-off. A total of 1/1 article found a significant increase in PSA with fatigue. A total of 0/1 articles found a significant increase or decrease in head-to-tibia shock attenuation with fatigue in jumping.

### 3.4. Overview of Fatigue Classification Performances

The model characteristics and classification performance for Type II articles can be found in Table 8. A total of 3/4 articles that investigated fatigue in walking used machine learning models, obtaining an accuracy ranging between 78% and 96%. A support vector machine (SVM) model was chosen in all three articles, in one case being the best-performing model compared to multiple different machine learning models. A total of 1/4 articles used multivariate forecast models to predict fatigue states. The best-performing model was an autoregressive integrated moving average (ARIMA) model with a mean absolute error (MAE) of 0.73 with respect to measured RPE values.

In SCTs, 1/1 article developed a model based on changes in body postures and kinetic energy to output a fatigue score. Correlation of the fatigue score with the RPE was quantified by Pearson’s r, being equal to 0.95 for males and equal to 0.70 for females.

In gym exercises, 1/1 article used machine learning models to estimate RPE values. Correlation between model outputs and the RPE was quantified by means of Pearson’s r, showing different results for different gym exercises: r = 0.89 for squats, r = 0.93 for jumping jacks, and r = 0.94 for corkscrew exercises. The machine learning models used were convolutional neural networks (CNN) and random forest (RF).

In running, 1/1 article developed a multiple linear regression time-to-exhaustion model. Pearson’s r was used to quantify correlation between the model’s output and RPE, with r being equal to 0.792.

Feature importance analyses were performed in 4/7 Type II articles across all the activities. In two articles, feature performance was performed before training the final model in order to improve model performance. In two articles, feature importance of the model was shown for the final model.

## 4. Discussion

The main scope of this literature review was to assess whether accelerometers are suitable sensors to identify physical fatigue in PE. In order to understand the real-life possibilities of fatigue detection in PE, we aimed to assess the capability of accelerometer-based parameters to straightforwardly estimate (traditional biomechanics) and assist in the detecting (machine learning) of physical fatigue. We found that identification of fatigue in PE using inertial sensors is mainly obtained by a straightforward comparison of biomechanical variables of interest or by training models that are validated by comparisons with physiological or subjective fatigue references.

Peak tibial and sacral acceleration were the most commonly sought outcomes. An increase in peak tibial or sacral acceleration with fatigue was found in 19/21 articles for running and jumping activities. However, segment acceleration was influenced by subject characteristics and the type of fatigue protocol (at particular speeds). Reporting these characteristics would facilitate the normalization of segmental acceleration results across articles and provide general, rather than individual, insight in its changes due to fatigue in PE. Other factors that were found to influence segment accelerations are training experience (elite vs. recreational), shoe type (prefabricated vs. custom-made sole), and running environment (treadmill vs. overground). This could explain the high variability across articles on PTA (4.5–24.6 g). Shock attenuation was found to increase with fatigue in 5/9 articles (running and jumping). While a high variability in biomechanical variables due to subject characteristics, number of subjects, and fatiguing protocols did not allow general conclusions, accelerometers were able to measure peak accelerations and shock attenuations at an individual level. Stride spatiotemporal parameters were also measured by accelerometers at an individual level in running, and significant changes were found in 5/16 articles. The low amount of articles that found a significant change in spatiotemporal parameters can be explained by the controlled constant speed in the majority of them.

Identification of physical fatigue using machine learning or other types of algorithms was performed in only 7 out of 39 articles. The accuracy of the models ranged between 78% and 96%, and Pearson’s correlation with RPE ranged between 0.79 and 0.95. Only two articles performed cross-validation (k-fold), suggesting that the validity of their results was specific for their subject population. Four articles provided further interpretation of their results by means of feature importance analysis, although the choice of features was either subjective or not specified. Changes in biomechanical variables found in the literature could provide a more objective choice of features for machine learning classifiers. While a generalized optimal method for PE activities was not found in this review, machine learning approaches succeeded in lower limb fatigue identification for each specific activity and were found to be less influenced by fatigue protocol characteristics than traditional biomechanics approaches.

Currently, a gold standard for the comprehensive measurement of physical fatigue in PE is missing. A total of 19/39 articles used Borg’s RPE as a fatigue reference or tried to predict and detect RPE levels. Borg’s RPE is a very practical scale to estimate fatigue, but it relates only to the mental components of fatigue. A total of 13/39 articles used cardiovascular or ventilatory parameters as fatigue references. They have the advantage of being objective metrics, but they are individual, often difficult to measure outside of a lab, and mostly related to the cardiovascular components of fatigue. Accelerometers have the potential to become extremely popular devices in the identification of physical fatigue in prolonged tasks out of a lab, but research protocols in the field of fatigue identification in human movement and PE are still too different from each other to draw general conclusions. Therefore, we provide five recommendations for future research in PE that could also be generally applied to human movement assessment (e.g., team sports, rehabilitation, and clinical practice) and may help the validation of accelerometers as a measurement system for the identification of physical fatigue.

### 4.1. Recommendations for Future Research in Physical Fatigue Identification Using Accelerometers

1. One of the aims of this review was to assess to what extent the fields of biomechanics and machine learning are useful to each other in fatigue identification. While a few articles developed fatigue models and assessed changes in biomechanics or feature importance [57,68,74], there is still uncertainty in the choice of model and machine learning biomechanical features. Developing consistent fatiguing protocols and reporting feature performance would improve biomechanical domain knowledge in machine learning studies, while automatic feature extraction techniques could also be used to improve model performance, as advocated by Halilaj et al. [29].
2. Biomechanical parameters of interest for fatigue estimation are influenced by many variables in PE. In this review, we identified sensor location, fatigue protocol, subject characteristics, training level, equipment, and environment. For example, accelerometer location on the distal part of the tibia causes an exposure to higher impacts and higher PTA than the proximal tibia. Accurate method descriptions would allow the proper comparison of biomechanical parameters and the generalization of results.
3. A subject being either in a fatigued or non-fatigued state is a simplified representation of more complex fatigue models that occur at the cardiovascular and neuromuscular levels [75]. An effort should be made in understanding and identifying fatigue development stages throughout a PE activity.
4. Fatigue detection, identification, or prediction with machine learning techniques should be generalized over subjects unless the objective is to train a subject-specific model [29]. Fatigue identification in PE is a large-scale problem and should be tackled with a subject-general model, since subject-specific models have limited scalability [76]. Leave-one-subject-out cross-validation should be used when trying to detect outcomes from different subjects, since it significantly helps model performance on new, unseen subjects [28].
5. Deep learning algorithms were not found in this review, although deep learning could be a promising technique to improve fatigue identification performance by reducing the need for feature engineering [29]. A possible explanation for the lack of deep learning algorithms could be the limited amount of data to train a deep leaning model with a good performance. The online sharing of data across research articles (also advocated by Gurchiek et al. [76]) could help developing a large dataset of accelerometer-based fatigue measurements in each PE activity.

### 4.2. Limitations

The main limitation of this study was the bias towards running activities (thirty out of thirty-nine articles). A possible explanation is the widespread popularity of running as a PE activity and the fact that its cyclical nature makes it an easy activity to analyze in research. However, the biomechanical outcomes of running can be applicable to other PE activities due to their quasi-cyclical nature [77], as well as more complex activities such as team sports. Team sports were not in the scope of this review, but running (and jumping, also evaluated in this review) are predominant component in many of them. Furthermore, extensive research in measuring running biomechanics using IMUs could be used by other sports that are starting to use a similar approach to monitor athletes in order to not repeat the same mistakes.

A second limitation in the analysis of accelerometer-based techniques was the assumption of similarity between accelerometer and IMU measurements. Although IMUs integrate data from gyroscopes and magnetometers, we assumed a neglectable impact on the measured outcomes of interest in our review. Further research would be needed to fully understand whether measurements performed with IMUs differ from measurements performed with simple accelerometers.

A third limitation was the lack of uniformity in fatigue protocols between the articles of this review. Fatigue protocols with different intensities (e.g., higher vs. lower speed) or different activities (e.g., running vs. squatting) can impact muscle activation differently. Single-muscle fatigue assessment was not in the scope of this review, but it has an impact on the onset of overall physical fatigue. Future studies should investigate the possibility to identify physical fatigue levels and link them to activity intensity. A standardization of fatigue protocols could also allow a meta-analysis of changes in biomechanical variables with fatigue in PE.

## 5. Conclusions

We aimed to assess whether accelerometer-based techniques could identify lower limb physical fatigue in PE. We found that changes in biomechanical parameters could be assessed at an individual level due to fatigue and that machine learning could help detect fatigue, but the link between machine learning and changes in biomechanics needs to be further investigated. Therefore, we formulated guidelines for future fatigue identification research using accelerometers. The aligning of fatigue protocols and online sharing of data could help validate biomechanical changes due to fatigue in the lower limbs and the large-scale deployment of accelerometers in physical fatigue assessment during PE.

## Figures and Tables

**Table 1:** Quality assessment items.

Type I Articles: Aim to Identify Changes Due to Fatigue in Lower Limb Biomechanics	Type II Articles: Aim to Identify, Predict, or Classify Fatigue States Based on Quantitative Biomechanical Features	
Quality assessment checklist items adapted from Downs and Black, 1998 [42]	Quality assessment checklist items adapted from Downs and Black, 1998 [42]	Potential score
1. Is the hypothesis, aim, or objective of the study clearly described?	1. Is the hypothesis, aim, or objective of the study clearly described?	0–1
2. Are the main outcomes to be measured clearly described in the Introduction or Methods section?	2. Are the main outcomes to be measured clearly described in the Introduction or Methods section?	0–1
3. Are the characteristics of the subject population clearly described?	3. Are the characteristics of the subject population clearly described?	0–1
4. Is the intervention (fatiguing protocol) clearly described?	4. Is the intervention (fatiguing protocol) clearly described?	0–1
5. Are the main findings of the study clearly described?	5. Does the study provide estimates of the random variability in the data for the main outcomes?	0–1
6. Does the study provide estimates of the random variability in the data for the main outcomes?	6. If any of the results of the study were based on “data dredging”, was this made clear?	0–1
7. Have actual probability values been reported (e.g., 0.035 rather than <0.05) for the main outcomes, except where the probability value is less than 0.001?	7. Were the main outcome measures used accurate (valid and reliable)?	0–1
	Quality assessment checklist items adapted from Luo et al., 2016 [43]	
8. If any of the results of the study were based on “data dredging”, was this made clear?	8. Was the prediction, classification, or identification problem defined?	0–1
9. Were the statistical tests used to assess the main outcomes appropriate?	9. Were the data prepared for model building?	0–1
10. Were the main outcome measures used accurate (valid and reliable)?	10. Was a classification, prediction, or identification model built?	0–1
11. Did the study have sufficient power to detect a clinically important effect where the probability value for a difference being due to chance is less than 5%?	11. Was the final model performance reported?	0–1

**Table 3:** Accelerometer placement: absolute number and percentage.

	Running	Jumping	Walking	SCT	Gym Exercises	Total
Tibia	22 (78%)	3 (75%)	3 (75%)	2 (100%)	1 (100%)	31 (79%)
Thigh	2 (7%)	0	0	2 (100%)	1 (100%)	5 (13%)
Sacrum	13 (46%)	1 (25%)	1 (25%)	2 (100%)	1 (100%)	18 (46%)
Foot	7 (25%)	0	0	2 (100%)	1 (100%)	10 (26%)
Total	28	4	4	2	1	

**Table 4:** Study protocol, data analysis, outcome(s) of interest, and quality assessment score.

	Measurement Protocol	Fatigue Protocol	Data Analysis	Outcome	Quality Assessment Score
**Authors [ref]**	**Activity (Setting)** **Speed (m/s)** **Duration (m)** **Rest Time (s)**	**Activity (Setting)** **Speed (m/s) or Number of Repetitions** **Duration (min) or Length (m)** **Fatigue Reference (mean RPE)** **Stopping Criteria**	**Measurement Points** **Amount of Data Per Point** **Filtering**	**Fatigue-Related Outcome(s)**	**DB: Adapted from Downs and Black Checklist [42]** **DBL: Adapted from Downs and Black [42] and Luo et al. [43] Checklists**
Abt et al. [44]	Running	Treadmill Speed NS (based on heart rate at VO2-max-based ventilatory threshold) 17.8 ± 5.7 min Heart rate at ventilatory threshold Run until exhaustion	2: start and end of FP 5 s /	PTA Acceleration reduction SA head–tibia	DB 11
Ameli et al. [19]	Stair climbing test: climbing up or down 10 steps over 90 s as fast as possible	1: Treadmill (1 rep) 2.78 m/s, 3.06 m/s, and 3.33 m/s 180 s 2: L-drill (3 reps) As fast as possible 30 s 3: Crunch + jumps (3 reps) As fast as possible 30 s 4: Sit to stand up + pushups (3 reps) As fast as possible 30 s RPE (1–10) Stopping criteria NS	2: before and after FP Subject-dependent: range of 0.00–11.50 laps /	Decrease in kinetic energy (KE) Decrease in twitch factor (TFA) Correlation between fatigue score (based on KE and TF) and RPE (Pearson’s r)	DBL 8
Arias-Torres et al. [45]	Walking: 200 m self-selected walk speed	Athletics track Speed NS (fastest sprint possible) / / 5.0% decrement of sprint time	2: before and after FP 100 m LP BW filter 5 Hz	Accuracy of the model Cohen’s Kappa Best feature subset	DBL 8
Bergmann et al. [46]	Stair climbing: ascending and descending a staircase 6 times	Recumbent ergometer reaching 80 revolutions per minute and back to 0 in the span of 12 s for 11 times 10 min RPE (1–10) Feeling uncomfortable	5: 2x before fatigue protocol, 1x after fatigue protocol (ergometer), 2x after fatigue protocol (stair climbing) / /	ROM (ankle, knee, thigh, trunk)	DB 8
Brahms et al. [47]	Running	200 m indoor track Speed: E: 4.5 ± 0.4; R: 3.5 ± 0.5 Duration: E: 19.8 ± 3.4; R: 26.2 ± 6.3 RPE E: 15.8 ± 1.1; R: 16.4 ± 1.4 Inability to maintain speed	3: beginning, middle and end of fatiguing run 1/3 of whole run (0–33% B; 34–66% M; 67–100% EN) LP BW filter 60 Hz (acc data) LP BW filter 15 Hz (stride and contact time)	PFA Stride time Stride length Contact time	DB 10
Butler et al. [48]	Running	Treadmill Self-selected training pace LA: 47 ± 24; HA: 52 ± 25 RPE and HR HR > 85% HR max; RPE > 16	2: beginning and end / /	PTA PTP (peak-to-peak tibial acceleration)	DB 11
Clansey et al. [23]	Running: 15 m overground trials x6 at 4.5 m/s	Treadmill LT speed at 3.5 mM blood lactate concentration 20 min (×2) RPE (6–20) Stopping criteria NS	3: Pre, mid, and post 2 fatiguing runs / Tibial accelerations downsampled to 60 Hz	PTA Step length	DB 10
Clermont et al. [49]	Running Marathon (4:26:23 ± 00:36:05 h:m:s)	Overground / 14 km (km 0 until km 14) / Stopping criteria NS	15: km 4–14, then from km 14 until end, 10 km (normal running segment), 2 km (14 fatigue segments) /	σ (biomechanical index) based on: step frequency, change in forward velocity, vertical oscillation, pelvic rotation, pelvic drop, contact time	DB 9
Coventry et al. [50]	Single-legged drop jumping (80% of maximum jump height)	Indoor Each cycle: DJ, CMJ, and 5 single-legged squats ~25 s RPE (1–10) Until unable to perform exercise	2: first and last cycle 1 cycle LP BW 15 Hz	PTA SA head–tibia (% and TF) Peak angular velocity	DB 10
Derrick et al. [51]	Running	Treadmill running 3.4 ± 0.4 m/s ~3200 m * ~15.7 min * / Until exhaustion	3: start, middle, end / 16 s	PTA SA head–tibia (% and TF) Stride length	DB 9
Encarnacion-Martinez et al. [52]	Treadmill running: 10 s ×3 at 3.89 m/s	Treadmill running 85% max aerobic speed 30 min RPE (6–20) Min of 17/20 RPE	2: pre and post 30 s /	PTA (max and total) SA head–tibia (% and TF)	DB 11
Garcia Perez et al. [53]	Running (treadmill and track, 400 m at 4 m/s)	Track running 85% of max effort 5 min run (3.81 ± 0.4 m/s) 30 min 6858 ± 720 m * / Stopping criteria NS	2: pre and post 3 strides /	PTA SA head–tibia (%) Tibial impact rate	DB 8
Hajifar et al. [17]	Walking for 8 m	Cycles of 16 squats 8 squats/min 2 min RPE RPE ≥ 17	Multiple: pre and post squats 8 m /	MAE of predictive model	DBL 10
Hardin et al. [54]	Running downhill	Treadmill running (downhill −12%) 30 min 3.4 m/s 6120 m * Creatine kinase variations Stopping criteria NS	6: every 5 min 10 strides /	PTA Stride frequency	DB 9
Hoenig et al. [55]	Running	Athletics track As fast as possible 5000 m VO2 max, lactate, and RPE after a previous 5 km run Stopping criteria NS	3: 500 m, 2500 m and 4500 m 150 strides /	LDS (quantified by largest Lyapunov exponent) sacrum, thorax, and foot	DB 10
Jiang et al. [56]	Gym exercises: squats, high knee jacks, and toe touches	Sets of squats, high knee jacks, and toe touches 5 repetitions 3–52 sets RPE Until exhaustion	All exercise repetitions Each repetition LP BW 20 Hz	RMSE and Pearson’s r between model and RPE	DBL 10
Karvekar et al. [57]	Walking for 8 meters	Cycles of 16 squats 8 squats/min 2 min RPE RPE ≥ 17	5: normal walking and 4 different RPE levels Sliding window of 70–80 data points LP BW 3 Hz	Accuracy and confusion matrix of model PTA	DBL 10
Lucas Cuevas et al. [58]	Running	Treadmill running Based on lactate threshold of 4.04 ± 0.36 m/s 15 min 3636 ± 324 m * Blood lactate concentration and RPE Stopping criteria NS	3: before, immediately after, and 2 min after 1 min 8th order LP Chebyshev type II 120 Hz (stop band ripple 40 Hz)	PTA SA head–tibia Stride frequency Stride length	DB 11
McGinnis et al. [59]	Vertical jumping: 4 maximal effort CMJs	4 maximal effort CMJs, obstacle course, and 4 maximal effort CMJs / RPE, HR, and performance decline HR = HR max after consecutive bouts, RPE = 10, or jump height decline to 70% of max effort	2: fatigue and non-fatigue 4 CMJs /	Vertical displacement sacrum Max vertical velocity sacrum Max vertical acceleration sacrum	DB 9
Meardon et al. [60]	Running	Indoor athletic track 3.49 ± 0.14 m/s 5.7 ± 0.9 km ~27.2 min * Percentage of HR max Running until exhaustion	3: beginning, middle, and end 0–33%, 34–67%, and 67–100% of each run 4th-order BP BW 0.9–50 Hz	Stride time (mean and long-range correlation)	DB 10
Mercer et al. [61]	Running (treadmill) 3.8 m/s, 8–10 min	Treadmill running Maximal effort graded exercise test (grade or speed increase every minute): 1.34 m/s (3%), 1.56 m/s (7.5%), 1.79 m/s (7.5%), 2.23 m/s (7.5%), and 2.45 m/s 3–5 min 1 min VO2, HR, and RPE Running until exhaustion	2: before and after 20 s /	PTA SA head–tibia PSD tibia Stride length	DB 8
Meyer et al. [62]	Running (5–10 km and 25–30 km, which correspond to the same section in the marathon)	Marathon running 42.1 km 5–10 km: 3.29 ± 0.35 m/s; 25–30 km: 3.16 ± 0.42 m/s / Stopping criteria NS	2: 5–10 km and 25–30 km 4012 ± 1250 LP BW 50 Hz	FSA Contact time Stride length	DB 10
Mizrahi_a_ [63], Mizrahi_b_ [64], Mizrahi_c_ [65]_,_ Mizrahi_d_ [66] et al.	Running	Treadmill running Speed at 5% exceeding AT 3.53 ± 0.19 m/s 30 min 6354 ± 342 m * Running until end of protocol Decline in PETCO2	7: every 5 min from start to end 20 s 8th-order LP BW 40 Hz	PTA PSA SA sacrum–tibia	DB 8,9,10,9
Moran et al. [18]	Drop jumping: 5 maximal effort drop jumps / / 15 s	Treadmill running 9.66 km/h (starting at 3% grade and increasing 1.5% every minute) 8.3 ± 2.4 min Less than 2 min RPE RPE ≥ 17	2: before and after 5 jumps /	PTA	DB 10
Morio et al. [67]	Running: run 11 ± 0.2 km/h for 3 min, treadmill, barefoot and shod (randomized)	Sledge ergometer exercise (25 bilateral rebounds) 266 ± 74 rebounds 9 ± 2.5 min Perceived muscle soreness Not reaching preset rebound of 80% on 10 consecutive rebounds	Running: 2 (pre and post) 3 min /	PTA	DB 8
Provot_a_ et al. [68]	Running	Treadmill running 3.75 m/s 38.5 ± 12.5 min 8662 ± 2812 m * RPE Unable to maintain speed	Whole run 38.5 ± 12.5 min /	Time to exhaustion model (RMSE and Pearson’s r)	DBL 11
Provot_b_ et al. [69]	Running	Treadmill running 3.75 m/s / RPE Running until exhaustion	10: every 5% of exhaustion level 30 strides /	CMD	DB 7
Reenalda_a_ et al. [22]	Running	Marathon running 3.61–4.08 m/s 42.2 km / Stopping criteria NS	4: 8 km, 18 km, 27 km, and 36 km 100 strides /	PSA Stride length Step frequency	DB 8
Reenalda_b_ et al. [27]	Running	Athletic track 4.39 ± 0.39 m/s 20 min 5268 ± 468 m * / Lactate threshold speed Stopping criteria NS	2: 3 min and 18 min 20 strides /	PTA PSA SA sacrum–tibia	DB 8
Ruder et al. [26]	Running	Marathon running Speed at 10 km: 3.41 ± 0.m/s; at 40 km: 2.92 ± 0.52 m/s 42.2 km / Stopping criteria NS	2: 5–10 km and 35–40 km 5 km /	PTA	DB 8
Sandrey et al. [70]	Vertical jumping: 3 maximal single leg vertical jumps	Triceps surae fatiguing protocol 23 heel raises per minute / / Pace no longer maintained or height not reached for 3 sequential heel raises	2: before and after 3 jumps	PTA	DB 11
Schutte_a_ et al. [71]	Running (speed 3.33 m/s)	Treadmill running Speed based on 3.2 km run at maximal effort 3.33 m/s 20.54 ± 6.90 min 4102 ± 1379 m * RPE Feeling unable to continue or RPE ≥ 17	2: beginning and end 20 steps LP BW 15 HZ	PSA Step frequency RMS of sacral acceleration	DB 10
Schutte_b_ et al. [25]	Running	Athletic track (outdoor) Speed based on 3.2 km run at maximal effort 3.92 m/s * 13.63 ± 1.86 min 3200 m RPE Stopping criteria NS	8: every 400 m 2229 ± 260 steps LP BW 50 Hz	PTA PSA SA sacrum–tibia Frequency domain: SA active phase magnitude and impact phase magnitude, signal power magnitude Contact time Step frequency	DB 10
Strohrmann et al. [72]	Running	Treadmill running Speed based on 85% 1 min run at max speed 2.5–4.94 m/s 45 min / Stopping criteria NS	2: beginning and end / /	COM displacement	DB 6
Verbitsky et al. [73]	Running	Treadmill running Speed at anaerobic threshold (NF 2.75 ± 0.48 m/s; F 2.76 ± 0.29 m/s) 30 min PETCO2 Decline in PETCO2	7: every 5 min 20 s /	PTA PSA	DB 8
Zhang et al. [74]	Walking in lab environment at self-preferred pace	Squatting 22 reps/min 52 ± 7 min MVE 60% of baseline MVE	2: before and after 6–7 gait cycles /	Accuracy, sensitivity, and specificity of model	DBL 10

* Indirectly estimated value based on data reported by the article; FP: fatiguing protocol; LP: low-pass; BW: Butterworth; E: elite; R: recreational; PFA: peak foot acceleration; PTA: peak tibial acceleration; PTP: peak-to-peak tibial acceleration; PSA: peak sacral acceleration; SA: shock attenuation; KE: kinetic energy; ROM: range of motion; TFA: twitch factor; TF: transfer function; LDS: local dynamic stability; PSD: power spectral density; FSA: foot strike angle; AT: anaerobic test; PETCO2: end-tidal carbon dioxide pressure; LT: lactate threshold; CMD: coefficient of multiple determination; MAE: mean absolute error; NS: not specified.

**Table 5:** Fatigue reference across fatiguing protocols: number of articles and percentages.

	Running	Jumping	Gym Exercises	Squats	Recumbent Ergometer	Triceps Surae	Total
RPE	12 (40%)	2 (100%)	1 (100%)	2 (66%)	2 (100%)	0	19 (49%)
HR parameters	5 (17%)	1 (50%)	0	0	0	0	6 (15%)
Ventilatory parameters	7 (23%)	0	0	0	0	0	7 (18%)
Other physiological parameters	3 (10%)	0	0	1 (33%)	0	0	4 (10%)
Total	30	2	1	3	2	1	

**Table 6:** Overview of biomechanical changes due to fatigue in running.

Authors [Ref]	Magnitude		Change	Fatigue Reference		Change in Fatigue Reference
	NF	F		NF	F	
Peak tibial acceleration (g)						
Abt et al. [44]	7.5 ± 1.1	7.7 ± 1.3	0.2 (*p* = 0.19)	/	/	/
Butler et al. [48]	MC 5.4 CT 4.5	MC 5.9 CT 4.6	MC 0.5 CT 0.1	/	/	/
Clansey et al. [23]	11.30 ± 2.15	11.79 ± 1.77	0.49 (*p* = 0.226)	RPE 11.8 ± 1.3	RPE 14.4 ± 1.5	2.6 * (*p* < 0.05)
Derrick et al. [51]	6.11 ± 0.96	7.38 ± 1.05	1.27 * (*p* < 0.05)	/	/	/
Garcia Perez et al. [53]	OG 24.6 ± 10.8 TM 15.3 ± 6.8	OG 22.2 ± 10.3 TM 17.2 ± 9.5	OG −2.4 TM 1.9	/	/	/
Hardin ¹ et al. [54]	10.6 ± 3.12	12.7 ± 3.95	2.1 * (*p* = 0.00)	/	/	/
Lucas Cuevas et al. [58]	CS 7.89 PS 8.13 CMS 7.69	CS 7.75 PS 8.59 CMS 7.96	CS −0.14 PS 0.46 CMS 0.27	/	RPE 14.34	/
Mercer et al. [61]	5.0 ± 1.6	5.3 ± 1.4	0.3	VO2 41.1 ± 2.7 HR 160 ± 10	47.9 ± 5.0 178 ± 10	6.8 * (*p* < 0.05) 18 * (*p* < 0.05)
Mizrahi_b,c_ et al. [64,65]	6.9 ± 2.9	11.1 ± 4.2	4.2 * (*p* = 0.03)	PETC02 43.9	37.2	−6.7 * (*p* = 0.045)
Morio et al. [67]	12.8 ± 3.9	18.9 ± 5.1	6.1 * (*p* = 0.005)	/	/	/
Reenalda_b_ et al. [27]	4.96 ± 1.57	5.33 ± 2.15	0.37 * (*p* < 0.05)	/	/	/
Ruder et al. [26]	11.94 ± 3.70	10.19 ± 3.40	−1.75 * (*p* < 0.01)	/	/	/
Verbitsky et al. [73]	9.80	15.68	5.88 * (*p* < 0.5)	PETC02 44.1	40.3	−3.8 * (*p* < 0.5)
Peak sacral acceleration (g)						
Mizrahi_a_ et al. [63]	2.41	3.50	1.09 * (*p* < 0.05)	PETC02 43.9	37.2	−6.7 * (*p* = 0.045)
Reenalda_a_ et al. [22]	3.63	4.14	0.51 * (*p* < 0.05)	/	/	/
Reenalda_b_ et al. [27]	2.51 ± 0.72	2.54 ± 0.62	0.03 (*p* = 0.338)	/	/	/
Schutte_a_ et al. [71]	1.39 ± 0.22	1.48 ± 0.21	0.09 (*p* = 0.007)	/	/	/
Peak foot acceleration (g)						
Brahms et al. [47]	E 20.1 ± 2.04 R 16.1 ± 3.87	E 20.8 ± 1.93 R 16.4 ± 3.57	E 0.7 R 0.3 * (*p* < 0.05)	/	E RPE 15.8, HR = 90.9 (%max) E RPE 16.4, HR = 92.3 (%max)	/
Shock attenuation (head–tibia)						
Abt et al. [44]	−14.2 ± 3.7 dB	−13.7 ± 3.1 dB	0.5 dB (*p* = 0.18)	/	/	/
Derrick et al. [51]	−13.6 ± 2.6 dB 74.5 ± 5.4%	−14.2 ± 2.7 dB 77.5 ± 4.1%	−0.6 dB 3.0% * (*p* < 0.05)	/	/	/
Encarnacion-Martinez et al. [52]	−54.73 ± 15.81 dB	−59.25 ± 16.12 dB	−4.52 dB * (*p* < 0.05)	/	RPE 17.6 ± 0.5	/
Garcia Perez et al. [53]	OG 82.1 ± 9.7% TM 75.5 ± 20.8%	OG 82.4 ± 8.7% TM 77.9 ± 13.9%	OG 0.3% TM 2.4%	/	/	/
Lucas Cuevas et al. [58]	CS 66.43% PS 67.37% CMS 65.78%	CS 66.82% PS 70.55% CMS 64.85%	CS 0.39% PS 3.18% CMS −0.93%	/	RPE 14.34	/
Mercer et al. [61]	−11.3 ± 2.7 dB	−9.8 ± 2.6 dB	2.5 dB * (*p* < 0.05)	VO2 41.1 ± 2.7 HR 160 ± 10	47.9 ± 5.0 178 ± 10	6.8 * (*p* < 0.05) 18 * (*p* < 0.05)
Shock attenuation (sacrum–tibia)						
Mizrahi_a_ et al. [63]	65.1%	74.2%	9.1% * (*p* < 0.05)	PETC02 43.9	37.2	−6.7 * (*p* = 0.045)
Reenalda_b_ et al. [27]	51.9 ± 16.2%	53.5 ± 15.0%	1.6% (*p* = 0.209)	/	/	/
Stride length (m)						
Brahms et al. [47]	E 3.01 ± 0.39 R 2.46 ± 0.34	E 3.01 ± 0.41 R 2.46 ± 0.33	E 0.0 R 0.0	/	E RPE 15.8, HR = 90.9 (%max) E RPE 16.4, HR = 92.3 (%max)	/
Derrick et al. [51]	2.43 ± 0.04	2.46 ± 0.04	0.03	/	/	/
Lucas Cuevas et al. [58]	CS 2.36 PS 2.36 CMS 2.36	CS 2.36 PS 2.36 CMS 2.37	CS 0.00 PS 0.00 CMS 0.01	/	RPE 14.34	/
Mercer et al. [61]	2.71 ± 0.15	2.75 ± 0.17	0.04	VO2 41.1 ± 2.7 HR 160 ± 10	47.9 ± 5.0 178 ± 10	6.8 * (*p* < 0.05) 18 * (*p* < 0.05)
Meyer et al. [62]	2.31 ± 0.18	2.23 ± 0.20	−0.08	/	/	/
Reenalda_a_ et al. [22]	2.56 ± 0.05	2.46 ± 0.10	0.10 * (*p* < 0.05)	/	/	/
Stride frequency						
Hardin ¹ et al. [54]	81.6 strides/min	82.8 strides/min	1.2 * strides/min (*p* = 0.01)	/	/	/
Lucas Cuevas et al. [58]	CS 1.41 strides/s PS 1.42 strides/s CMS 1.41 strides/s	CS 1.42 strides/s PS 1.42 strides/s CMS 1.37 strides/s	CS 0.01 strides/s PS 0.0 strides/s CMS −0.04 strides/s	/	RPE 14.34	/
Stride time (msec)						
Brahms et al. [47]	E 698 ± 46 R 710 ± 40	E 696 ± 46 R 710 ± 39	E −2 R 0	/	E RPE 15.8, HR = 90.9 (%max) E RPE 16.4, HR = 92.3 (%max)	/
Meardon et al. [60]	700 ± 12	700 ± 12	0	/	/	/
Step length (m)						
Clansey et al. [23]	1.70 ± 0.05	1.69 ± 0.06	−0.1 (*p* = 0.698)	RPE 11.8 ± 1.3	RPE 14.4 ± 1.5	2.6 * (*p* < 0.05)
Step frequency (steps/min)						
Reenalda_a_ et al. [22]	176.56 ± 3.18	177.68 ± 4.97	1.12 * (*p* < 0.05)	/	/	/
Schutte_a_ et al. [71]	162.44 ± 7.54	162.88 ± 8.15	0.44 (*p* = 0.74)			
Contact time (msec)						
Brahms et al. [47]	E 147 ± 8 R 171 ± 16	E 148 ± 8 R 172 ± 16	E 1 R 1	/	E RPE 15.8, HR = 90.9 (%max) E RPE 16.4, HR = 92.3 (%max)	/
Meyer et al. [62]	214 ± 28	228 ± 37	14 * (*p* < 0.05)	/	/	/
Foot strike angle (deg)						
Meyer et al. [62]	12.35 ± 1.88	10.36 ± 1.65	−1.99 * (*p* < 0.05)	/	/	/

¹ Downhill running. * indicates significant difference. NF: non-fatigued condition, F: fatigued condition, MC: motion control shoe, CT: cushioning shoe, CS: control shoe, PS: pre-fabricated shoe, CMS: custom-made shoe, OG: overground, TM: treadmill, E: Elite, R: Recreational, RPE: rate of perceived exertion (scale 6–20), VO2: oxygen consumption (ml·kg^−1^·min^−1^), HR: heart rate (beat·min^−1^), PETCO2: end-tidal carbon dioxide pressure (Torr).

**Table 7:** Overview of biomechanical changes due to fatigue in jumping articles.

Article	Magnitude		Change in Magnitude	Fatigue Reference		Change in Fatigue Reference
	NF	F		NF	F	
Peak tibial acceleration (g)						
Coventry et al. [50]	13.4 ± 4.7	12.2 ± 1.7	1.2 (*p* = 0.420)	RPE = 6.56 ± 0.98	19.72 ± 0.84	13.16 * (*p* < 0.001)
Moran et al. [18]	15.8 (30 cm jump) 22.6 (50 cm jump)	19.6 (30 cm jump) 23.8 (50 cm jump)	3.8 * (*p* < 0.05) 1.2	/	/	/
Sandrey et al. [70]	5.19 ± 1.61 (take-off) 5.82 ± 1.70 (landing)	5.34 ± 1.58 (take-off) 6.65 ± 1.96 (landing)	0.15 (take-off, *p* = 0.19) 0.83 * (landing, *p* < 0.01)	/	/	/
Peak sacral acceleration (g)						
McGinnis et al. [59]	/	/	0.15 * (*p* = 0.03)	/	/	/
Shock attenuation (head–tibia)						
Coventry et al. [50]	−12.7 ± 3.7 dB 70.1 ± 4.6%	−14.7 ± 3.7 dB 70.8 ± 7.8%	−1.0 dB (0.416) 0.7% (*p* = 0.839)	RPE = 6.56 ± 0.98	19.72 ± 0.84	13.16 * (*p* < 0.001)

* indicates significant difference. NF: non-fatigued condition, F: fatigued condition, RPE: rate of perceived exertion (scale 6–20).

**Table 8:** Fatigue classification and prediction performance across all PE activities.

Authors [Ref]	Activity and Fatigue Protocol	N° of Subjects	Measurement Points	Algorithms or Model Used	Validation Type	Fatigue Reference	Outcomes
Ameli et al. [19]	SCT (Running + gym exercises FP)	20	2: before and after FP	Gaussian mixture model for changes in body posture and kinetic energy	/	RPE	Pearson’s r: 0.95 (males) and 0.7 (females)
Arias-Torres et al. [45]	Walking (Running FP)	9	2: before and after FP	LDA, CART, SVM, KNN, RF, NB	k-fold CV (k = 10)	Decrease in performance	Accuracy: 0.78 SVM Feature importance analysis
Hajfar et al. [17]	Walking (Squatting FP)	24	Multiple: pre and post squats	Multivariate forecasting models (Naïve, AR, VAR, ARIMA, VECM)	/	RPE	MAE < 1.24 ARIMA
Jiang et al. [56]	Gym exercises (Sets of squats, high knee jacks, and toe touches)	14	1 per repetition	CNN and RF	/	RPE	Pearson’s r: 89%, 93%, and 94% correlation for squat, jacks, and corkscrew exercises, respectively Feature importance analysis
Karvekar et al. [57]	Walking (Squatting FP)	24	5 throughout FP	SVM	/	RPE	Accuracy and confusion matrix of model: 91% Feature importance analysis
Provot_a_ et al. [68]	Running	10	Whole run	Time to exhaustion model (multiple linear regression)	/	RPE	Pearson’s r: 0.792 Feature importance analysis
Zhang et al. [74]	Walking (Squatting FP)	17	2: before and after FP	SVM	k-fold CV (k = 5)	Decrease in performance	Accuracy: 96%

FP: fatigue protocol, LDA: linear discriminant analysis, CART: classification and regression tree, SVM: support vector machine, KNN: k-nearest neighbors, RF: random forest, CNN: convolutional neural networks, NB: naïve Bayes, AR: autoregressive, VAR: vector autoregressive, ARIMA: autoregressive integrated moving average, VECM: vector error correction model, MAE: mean absolute error, CV: cross-validation.

## Supplementary Materials

The following are available online at https://www.mdpi.com/article/10.3390/s22083008/s1: Table S1: PRISMA 2020 Checklist; Figure S1: Flow of systematic review process according to PRISMA diagram after first search (August 2020); Figure S2: Flow of systematic review process according to PRISMA diagram after second search (May 2021); Table S2: Full Quality assessment for Type I articles; Table S3: Full Quality assessment for Type II articles.

## Appendix A

**Table A1:** Keywords used for each database search.

Embase.com (Last accessed on 31 May 2021)	(‘fatigue’/de OR ‘muscle fatigue’/de OR exhaustion/de OR (fatigue* OR exhaust* OR exertion* OR tired*):ab,ti) AND (‘gait’/de OR ‘physical activity’/de OR ‘walking’/de OR ‘lower limb’/exp OR ‘running’/de OR jogging/de OR ‘daily life activity’/exp OR ‘kinetics’/de OR ‘motion’/de OR ‘biomechanics’/de OR treadmill/de OR exercise/de OR ‘runner’/de OR ‘marathon runner’/de OR ‘motion analysis system’/de OR ‘motor activity’/de OR ‘exercise test’/exp OR (gait OR walking OR running OR jogging OR (lower NEAR/3 (limb* OR extermit*)) OR knee OR knees OR hip OR hips OR ankle* OR foot OR feet OR leg OR legs OR thigh* OR work-task* OR (daily NEAR/3 (life OR living) NEAR/6 activit*) OR ((physical* OR Motor*) NEAR/3 activit*) OR stride OR kinetic* OR motion OR biomechanic* OR treadmill* OR exercise* OR work OR workplace OR worker* OR stand OR standing):ab,ti) AND (‘inertial measurement unit’/de OR ‘inertial measurement unit sensor’/de OR ‘accelerometer’/de OR ‘gyroscope’/de OR ‘accelerometry’/de OR (‘acceleration’/de AND (‘smartphone’/de OR ‘mobile application’/de)) OR (((inertial*) NEAR/3 measur*) OR acceleromet* OR gyroscope* OR imu OR imus OR immu OR immus OR ((inertial OR body OR wearable*) NEXT/1 sens*) OR xsens OR x-sens OR jerk OR ((smartphone* OR app OR mobile-application*) NEAR/3 (accelerat* OR Measure*))):Ab,ti) NOT ([conference abstract]/lim AND [1800–2018]/py) AND [English]/lim NOT ([animals]/lim NOT [humans]/lim)
Medline ALL Ovid (Last accessed on 31 May 2021)	(Fatigue/ OR Muscle Fatigue/ OR (fatigue* OR exhaust* OR exertion* OR tired*).ab,ti.) AND (Gait/ OR Walking/ OR exp Lower Extremity/ OR Running/ OR Jogging/ OR Activities of Daily Living / OR Kinetics/ OR Motion/ OR Biomechanical Phenomena / OR Exercise Test / OR Exercise/ OR Motor Activity/ OR (gait OR walking OR running OR jogging OR (lower ADJ3 (limb* OR extermit*)) OR knee OR knees OR hip OR hips OR ankle* OR foot OR feet OR leg OR legs OR thigh* OR work-task* OR (daily ADJ3 (life OR living) ADJ6 activit*) OR ((physical* OR Motor*) ADJ3 activit*) OR stride OR kinetic* OR motion OR biomechanic* OR treadmill* OR exercise* OR work OR workplace OR worker* OR stand OR standing).ab,ti.) AND (Accelerometry/ OR (Acceleration/ AND (Smartphone/ OR Mobile Applications/)) OR (((inertial*) ADJ3 measur*) OR acceleromet* OR gyroscope* OR imu OR imus OR immu OR immus OR ((inertial OR body OR wearable*) ADJ sens*) OR xsens OR x-sens OR jerk OR ((smartphone* OR app OR mobile-application*) ADJ3 (accelerat* OR Measure*))).ab,ti.) AND english.la. NOT (exp animals/ NOT humans/)
CINAHL EBSCOHost (Last accessed on 31 May 2021)	(MH Fatigue OR MH Muscle Fatigue OR ti(fatigue* OR exhaust* OR exertion* OR tired*) OR ab(fatigue* OR exhaust* OR exertion* OR tired*)) AND (MH Gait OR MH Walking OR MH Lower Extremity+ OR MH Running OR MH Jogging OR MH Activities of Daily Living OR MH Kinetics OR MH Motion OR MH Exercise Test OR MH Exercise OR MH Motor Activity OR ti(gait OR walking OR running OR jogging OR (lower N2 (limb* OR extermit*)) OR knee OR knees OR hip OR hips OR ankle* OR foot OR feet OR leg OR legs OR thigh* OR work-task* OR (daily N2 (life OR living) N5 activit*) OR ((physical* OR Motor*) N2 activit*) OR stride OR kinetic* OR motion OR biomechanic* OR treadmill* OR exercise* OR work OR workplace OR worker* OR stand OR standing) OR ab(gait OR walking OR running OR jogging OR (lower N2 (limb* OR extermit*)) OR knee OR knees OR hip OR hips OR ankle* OR foot OR feet OR leg OR legs OR thigh* OR work-task* OR (daily N2 (life OR living) N5 activit*) OR ((physical* OR Motor*) N2 activit*) OR stride OR kinetic* OR motion OR biomechanic* OR treadmill* OR exercise* OR work OR workplace OR worker* OR stand OR standing)) AND (MH Accelerometry OR (MH “Acceleration (Mechanics)” AND (MH Smartphone OR MH Mobile Applications)) OR TI(((inertial*) N2 measur*) OR acceleromet* OR gyroscope* OR imu OR imus OR immu OR immus OR ((inertial OR body OR wearable*) N1 sens*) OR xsens OR x-sens OR jerk OR ((smartphone* OR app OR mobile-application*) N2 (accelerat* OR Measure*))) OR AB(((inertial*) N2 measur*) OR acceleromet* OR gyroscope* OR imu OR imus OR immu OR immus OR ((inertial OR body OR wearable*) N1 sens*) OR xsens OR x-sens OR jerk OR ((smartphone* OR app OR mobile-application*) N2 (accelerat* OR Measure*)))) AND LA(English) NOT (MH animals+ NOT MH humans+)
Web of science Core Collection (Last accessed on 31 May 2021)	TS = (((fatigue* OR exhaust* OR exertion* OR tired*)) AND ((gait OR walking OR running OR jogging OR (lower NEAR/2 (limb* OR extermit*)) OR knee OR knees OR hip OR hips OR ankle* OR foot OR feet OR leg OR legs OR thigh* OR work-task* OR (daily NEAR/2 (life OR living) NEAR/5 activit*) OR ((physical* OR Motor*) NEAR/2 activit*) OR stride OR kinetic* OR motion OR biomechanic* OR treadmill* OR exercise* OR work OR workplace OR worker* OR stand OR standing)) AND ((((inertial*) NEAR/2 measur*) OR acceleromet* OR gyroscope* OR imu OR imus OR immu OR immus OR ((inertial OR body OR wearable*) NEAR/1 sens*) OR xsens OR x-sens OR jerk OR ((smartphone* OR app OR mobile-application*) NEAR/2 (accelerat* OR Measure*))))) AND DT = (article) AND LA = (english)

**Table A2:** Eligibility criteria (EC) during title and abstract screening phase. Articles were excluded if the title or abstract suggests that:

EC 1.1	The study population is formed by non-healthy subjects, either at the time of the study or in rehabilitation
EC 1.2	The study population average age is lower than 18 years or higher than 70 years
EC 1.3	The activities performed are not a sport, ADL, or working task involving moving or standing
EC 1.4	The study does not include one of the three following sensors: accelerometer, gyroscope, or magnetometer, or does not include IMUs
EC 1.5	The study does not include at least a sensor on the lower limbs
EC 1.6	The article is not in English
EC 1.7	The article is a conference paper

**Table A3:** Eligibility criteria (EC) during full-text screening phase. Articles were excluded if:

EC 2.1	No primary data were collected
EC 2.2	The study does not focus on individual physical exercise tasks
EC 2.3	The study performed measurements spreading over multiple days
EC 2.4	The study protocol requires subject to perform power-assisted body movements
EC 2.5	The study does not include kinetic or kinematic parameters for the lower limbs
EC 2.6	The study lacks a fatigue inducement protocol, in particular: For protocols involving running: no running-induced fatigue (minimum of no-stop 3 km running if not stated) For protocols involving static physical exercise or walking: protocol-induced fatigue and exhaustion
EC 2.7	The study protocol includes an accelerometer or IMU with a sampling frequency <60 Hz
EC 2.8	The study is a case study (1 subject only)
