# Supplementary material for: Accelerometer-Based Identification of Fatigue in the Lower Limbs during Cyclical Physical Exercise: A Systematic Review
Source: Sensors (Basel). 2022 Apr 14;22(8):3008. doi: 10.3390/s22083008 (PMC9025833; doi:10.3390/s22083008)
Supplement: Supplementary file 1 [file sensors-22-03008-s001.zip › sensors-1670607-supplementary.pdf]

Table S1: PRISMA 2020 Checklist

Section and Topic	Item #	Checklist item	Location where item is reported
**TITLE**			
Title	1	Identify the report as a systematic review.	1
**ABSTRACT**			
Abstract	2	See the PRISMA 2020 for Abstracts checklist.	1
**INTRODUCTION**			
Rationale	3	Describe the rationale for the review in the context of existing knowledge.	1-3
Objectives	4	Provide an explicit statement of the objective(s) or question(s) the review addresses.	2-3
**METHODS**			
Eligibility criteria	5	Specify the inclusion and exclusion criteria for the review and how studies were grouped for the syntheses.	3
Information sources	6	Specify all databases, registers, websites, organisations, reference lists and other sources searched or consulted to identify studies. Specify the date when each source was last searched or consulted.	3
Search strategy	7	Present the full search strategies for all databases, registers and websites, including any filters and limits used.	3
Selection process	8	Specify the methods used to decide whether a study met the inclusion criteria of the review, including how many reviewers screened each record and each report retrieved, whether they worked independently, and if applicable, details of automation tools used in the process.	3-5
Data collection process	9	Specify the methods used to collect data from reports, including how many reviewers collected data from each report, whether they worked independently, any processes for obtaining or confirming data from study investigators, and if applicable, details of automation tools used in the process.	3
Data items	10a	List and define all outcomes for which data were sought. Specify whether all results that were compatible with each outcome domain in each study were sought (e.g. for all measures, time points, analyses), and if not, the methods used to decide which results to collect.	5
	10b	List and define all other variables for which data were sought (e.g. participant and intervention characteristics, funding sources). Describe any assumptions made about any missing or unclear information.	5
Study risk of bias assessment	11	Specify the methods used to assess risk of bias in the included studies, including details of the tool(s) used, how many reviewers assessed each study and whether they worked independently, and if applicable, details of automation tools used in the process.	3
Effect measures	12	Specify for each outcome the effect measure(s) (e.g. risk ratio, mean difference) used in the synthesis or presentation of results.	5
Synthesis methods	13a	Describe the processes used to decide which studies were eligible for each synthesis (e.g. tabulating the study intervention characteristics and comparing against the planned groups for each synthesis (item #5)).	5-6
	13b	Describe any methods required to prepare the data for presentation or synthesis, such as handling of missing summary statistics, or data conversions.	6
	13c	Describe any methods used to tabulate or visually display results of individual studies and syntheses.	na

Section and Topic	Item #	Checklist item	Location where item is reported
	13d	Describe any methods used to synthesize results and provide a rationale for the choice(s). If meta-analysis was performed, describe the model(s), method(s) to identify the presence and extent of statistical heterogeneity, and software package(s) used.	3-5
	13e	Describe any methods used to explore possible causes of heterogeneity among study results (e.g. subgroup analysis, meta-regression).	na
	13f	Describe any sensitivity analyses conducted to assess robustness of the synthesized results.	na
Reporting bias assessment	14	Describe any methods used to assess risk of bias due to missing results in a synthesis (arising from reporting biases).	6
Certainty assessment	15	Describe any methods used to assess certainty (or confidence) in the body of evidence for an outcome.	6
**RESULTS**			
Study selection	16a	Describe the results of the search and selection process, from the number of records identified in the search to the number of studies included in the review, ideally using a flow diagram.	4
	16b	Cite studies that might appear to meet the inclusion criteria, but which were excluded, and explain why they were excluded.	4
Study characteristics	17	Cite each included study and present its characteristics.	6-18
Risk of bias in studies	18	Present assessments of risk of bias for each included study	19
Results of individual studies	19	For all outcomes, present, for each study: (a) summary statistics for each group (where appropriate) and (b) an effect estimate and its precision (e.g. confidence/credible interval), ideally using structured tables or plots.	6-25
Results of syntheses	20a	For each synthesis, briefly summarise the characteristics and risk of bias among contributing studies.	19-25
	20b	Present results of all statistical syntheses conducted. If meta-analysis was done, present for each the summary estimate and its precision (e.g. confidence/credible interval) and measures of statistical heterogeneity. If comparing groups, describe the direction of the effect.	19-25
	20c	Present results of all investigations of possible causes of heterogeneity among study results.	19-20
	20d	Present results of all sensitivity analyses conducted to assess the robustness of the synthesized results.	na
Reporting biases	21	Present assessments of risk of bias due to missing results (arising from reporting biases) for each synthesis assessed.	na
Certainty of evidence	22	Present assessments of certainty (or confidence) in the body of evidence for each outcome assessed.	19-25
**DISCUSSION**			
Discussion	23a	Provide a general interpretation of the results in the context of other evidence.	25-26
	23b	Discuss any limitations of the evidence included in the review.	27
	23c	Discuss any limitations of the review processes used.	27
	23d	Discuss implications of the results for practice, policy, and future research.	26
**OTHER INFORMATION**			
Registration and protocol	24a	Provide registration information for the review, including register name and registration number, or state that the review was not registered.	na

Section and Topic	Item #	Checklist item	Location where item is reported
	24b	Indicate where the review protocol can be accessed, or state that a protocol was not prepared.	na
	24c	Describe and explain any amendments to information provided at registration or in the protocol.	na
Support	25	Describe sources of financial or non-financial support for the review, and the role of the funders or sponsors in the review.	27
Competing interests	26	Declare any competing interests of review authors.	28
Availability of data, code and other materials	27	Report which of the following are publicly available and where they can be found: template data collection forms; data extracted from included studies; data used for all analyses; analytic code; any other materials used in the review.	na

Figure S1: Flow of systematic review process according to PRISMA diagram after first search (August 2020). *Records excluded via exclusion criteria in Table A2. ** Records excluded via exclusion criteria in Table A3

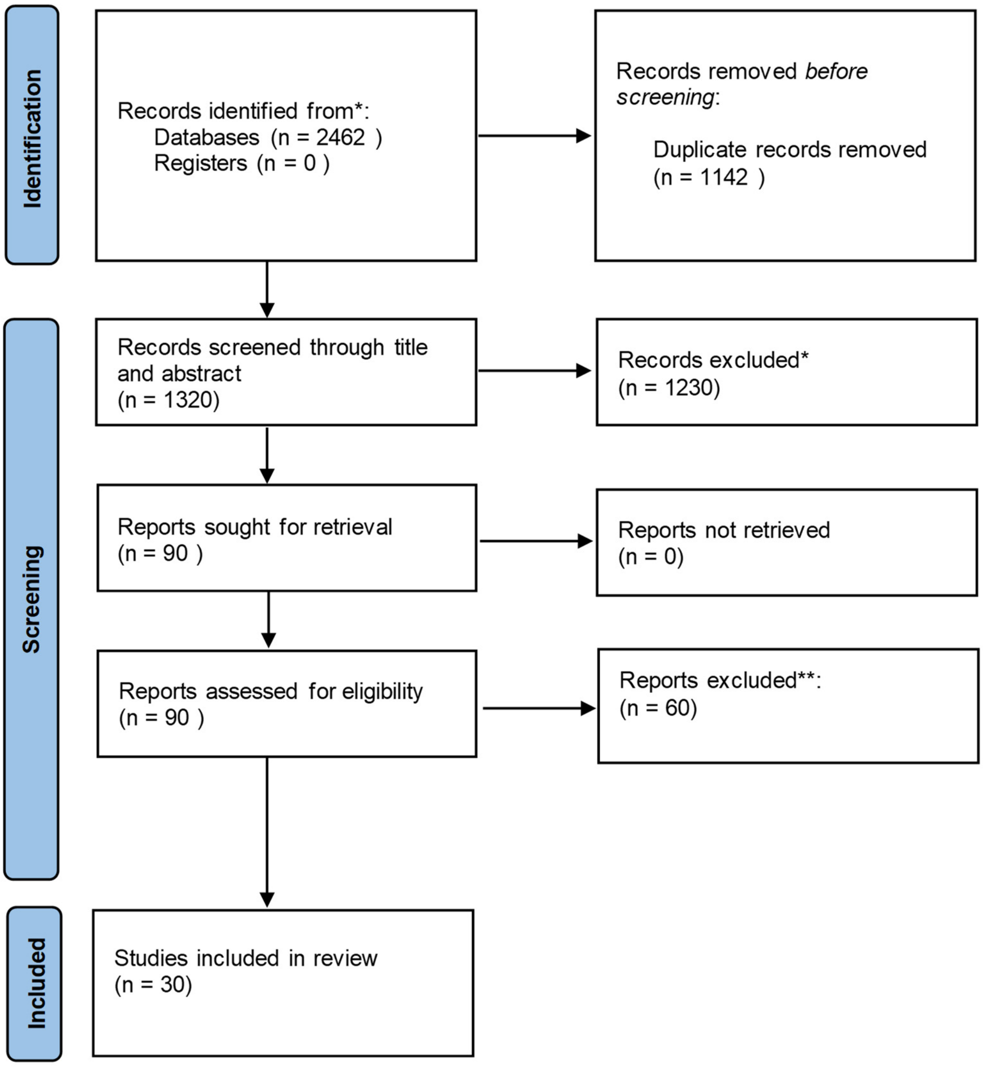

Figure S2: Flow of systematic review process according to PRISMA diagram after second search (May 2021). *Records excluded via exclusion criteria in Table A2. ** Records excluded via exclusion criteria in Table A3.

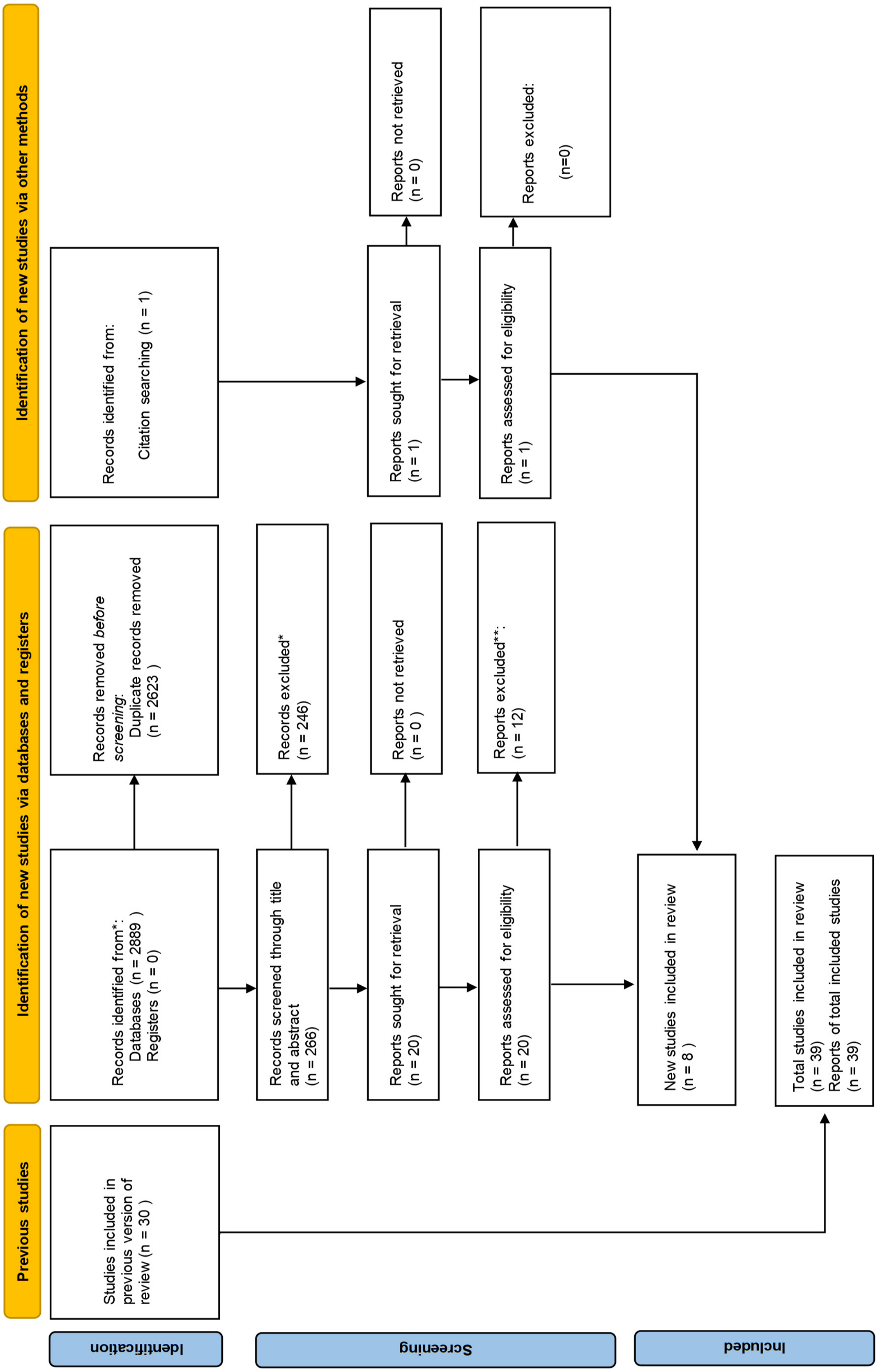

Table S2: Full Quality assessment for Type I studies. Score is equal to one when it fulfils the requirements from Downs and Black [1], zero otherwise.

**Quality assessment checklist items adapted from Downs and Black** [1]
1. Is the hypothesis, aim or objective of the study clearly described?
2. Are the main outcomes to be measured clearly described in the Introduction or Methods section?
3. Are the characteristics of the subject population clearly described?
4. Is the intervention (fatiguing protocol) clearly described?
5. Are the main findings of the study clearly described?
6. Does the study provide estimates of the random variability in the data for the main outcomes?
7. Have actual probability values been reported (e.g. 0.035 rather than <0.05) for the main outcomes except where the probability value is less than 0.001?
8. If any of the results of the study were based on “data dredging”, was this made clear?
9. Were the statistical tests used to assess the main outcomes appropriate?
10. Were the main outcome measures used accurate (valid and reliable)?
11. Did the study have sufficient power to detect a clinically important effect where the probability value for a difference being due to chance is less than 5%?

	Checklist items for Type I studies adapted from Downs and Black [1]											Final score
Author [Ref]	#1	#2	#3	#4	#5	#6	#7	#8	#9	#10	#11	
Abt et al. [2]	1	1	1	1	1	1	1	1	1	1	1	11
Bergmann et al. [3]	1	1	0	1	1	0	1	1	1	1	0	8
Brahms et al. [4]	1	1	1	1	1	1	1	1	1	1	0	10
Butler et al. [5]	1	1	1	1	1	1	1	1	1	1	1	11
Clansey et al. [6]	1	1	1	1	1	1	1	1	1	1	0	10
Clermont et al. [7]	1	1	1	0	1	0	1	1	1	1	1	9
Coventry et al. [8]	1	1	1	1	1	1	1	1	1	1	0	10
Derrick et al. [9]	1	1	1	0	1	1	1	1	1	1	0	9
Encarnacion-Martinez et al. [10]	1	1	1	1	1	1	1	1	1	1	1	11
Garcia Perez et al. [11]	1	1	0	1	1	1	0	1	1	1	0	8
Hardin et al. [12]	1	1	0	0	1	1	1	1	1	1	1	9
Hoenig et al. [13]	1	1	1	0	1	1	1	1	1	1	1	10
Lucas Cuevas et al. [14]	1	1	1	1	1	1	1	1	1	1	1	11
McGinnis et al. [15]	1	1	0	1	1	1	1	1	1	1	0	9
Meardon et al. [16]	1	1	1	1	1	1	1	1	1	1	0	10
Mercer et al. [17]	1	1	0	1	1	1	0	1	1	1	0	8
Meyer et al. [18]	1	1	1	0	1	1	1	1	1	1	1	10
Mizrahi$_a$ et al. [19]	1	1	1	1	1	1	0	1	0	1	0	8
Mizrahi$_b$ et al.[20]	1	1	1	1	1	1	0	1	1	1	0	9
Mizrahi$_c$ et al.[21]	1	1	1	1	1	1	1	1	1	1	0	10
Mizrahi$_d$ et al.[22]	1	1	1	1	1	1	0	1	1	1	0	9
Moran et al. [23]	1	1	1	1	1	1	1	1	1	1	0	10
Morio et al. [24]	1	1	0	1	1	0	1	1	1	1	0	8
Provot$_b$ et al. [25]	1	1	0	1	0	0	1	1	1	1	0	7
Reenalda$_a$ et al. [26]	1	1	0	0	1	1	1	1	1	1	0	8
Reenalda$_b$ et al. [27]	1	1	1	0	1	1	0	1	1	1	0	8
Ruder et al. [28]	1	1	1	0	1	1	0	1	1	1	0	8
Sandrey et al. [29]	1	1	1	1	1	1	1	1	1	1	1	11
Schutte$_a$ et al. [30]	1	1	1	1	1	1	1	1	1	1	0	10
Schutte$_b$ et al. [31]	1	1	1	0	1	1	1	1	1	1	1	10
Strohrmann et al. [32]	1	0	0	0	1	0	1	1	1	1	0	6
Verbitsky et al. [33]	1	1	1	1	1	1	0	1	0	1	0	8

Table S3: Full Quality assessment for Type II studies. Score is equal to one when it fulfils the requirements from Downs and Black [1] and Luo et al. [34] , zero otherwise.

**Quality assessment checklist items adapted from Downs and Black** [1]
1. Is the hypothesis, aim or objective of the study clearly described?
2. Are the main outcomes to be measured clearly described in the Introduction or Methods section?
3. Are the characteristics of the subject population clearly described?
4. Is the intervention (fatiguing protocol) clearly described?
6. Does the study provide estimates of the random variability in the data for the main outcomes?
8.If any of the results of the study were based on "data dredging", was this made clear?
11. Were the main outcome measures used accurate (valid and reliable)?
**Quality assessment checklist items adapted from Luo et al.** [34]
1. Was the prediction/classification/identification problem defined?
2. Were the data prepared for model building?
3. Was a classification/prediction/identification model built?
4. Was the final model performance reported?

	Checklist items for Type II studies adapted from Downs and Black, 1998 [1]							Checklist items for Type II studies adapted from Luo et al., 2016 [34]				Final score
Author, year	#1	#2	#3	#4	#6	#8	#11	#1	#2	#3	#4	
Ameli et al. [35]	1	1	1	1	0	1	0	1	0	1	1	8
Arias-Torres et al. [36]	1	1	0	0	1	1	0	1	1	1	1	8
Hajfar et al. [37]	1	1	0	1	1	1	1	1	1	1	1	10
Jiang et al. [38]	1	1	0	1	1	1	1	1	1	1	1	10
Karvekar et al. [39]	1	1	1	1	1	1	0	1	1	1	1	10
Provot$_a$ et al. [40]	1	1	1	1	1	1	1	1	1	1	1	11
Zhang et al. [41]	1	1	1	1	0	1	1	1	1	1	1	10

References

[1] S. H. Downs and N. Black, "The feasibility of creating a checklist for the assessment of the methodological quality both of randomised and non-randomised studies of health care interventions," *J Epidemiol Community Heal.*, vol. 52, pp. 377–384, 1998.

[2] J. P. Abt, T. C. Sell, Y. Chu, M. Lovalekar, R. G. Burdett, and S. M. Lephart, "Running kinematics and shock absorption do not change after brief exhaustive running," *J. Strength Cond. Res.*, vol. 25, no. 6, pp. 1479–1485, 2011, doi: 10.1519/JSC.0b013e3181ddfcf8.

[3] J. H. M. Bergmann, I. C. H. Smith, and R. E. Mayagoitia, "Using a body sensor network to measure the effect of fatigue on stair climbing performance," *Physiol. Meas.*, vol. 33, no. 2, pp. 287–296, 2012, doi: 10.1088/0967-3334/33/2/287.

[4] C. M. Brahms, Y. Zhao, D. Gerhard, and J. M. Barden, "Long-range correlations and stride pattern variability in recreational and elite distance runners during a prolonged run," *Gait Posture*, vol. 92, no. July, pp. 487–492, 2022, doi: 10.1016/j.gaitpost.2020.08.107.

[5] R. J. Butler, J. Hamill, and I. Davis, "Effect of footwear on high and low arched runners' mechanics during a prolonged run," *Gait Posture*, vol. 26, no. 2, pp. 219–225, 2007, doi: 10.1016/j.gaitpost.2006.09.015.

[6] A. C. Clansey, M. Hanlon, E. S. Wallace, and M. J. Lake, "Effects of fatigue on running mechanics associated with tibial stress fracture risk," *Med. Sci. Sports Exerc.*, vol. 44, no. 10, pp. 1917–1923, Oct. 2012, doi: 10.1249/MSS.0b013e318259480d.

[7] C. A. Clermont, L. C. Benson, W. B. Edwards, B. A. Hettinga, and R. Ferber, "New considerations for wearable technology data: Changes in running biomechanics during a marathon," *J. Appl. Biomech.*, vol. 35, no. 6, pp. 401–409, 2019, doi: 10.1123/jab.2018-0453.

[8] E. Coventry, K. M. O'Connor, B. A. Hart, J. E. Earl, and K. T. Ebersole, "The effect of lower extremity fatigue on shock attenuation during single-leg landing," *Clin. Biomech.*, vol. 21, no. 10, pp. 1090–1097, 2006, doi: 10.1016/j.clinbiomech.2006.07.004.

[9] T. R. Derrick, D. Dereu, and S. P. Mclean, "Impacts and kinematic adjustments during an exhaustive run," *Med. Sci. Sports Exerc.*, vol. 34, no. 6, pp. 998–1002, 2002, doi: 10.1097/00005768-200206000-00015.

[10] A. Encarnación-Martínez, R. Sanchis-Sanchis, P. Pérez-Soriano, and A. García-Gallart, "Relationship between muscular extensibility, strength and stability and the transmission of impacts during fatigued running," *Sport. Biomech.*, vol. 00, no. 00, pp. 1–17, 2020, doi: 10.1080/14763141.2020.1797863.

[11] J. A. García-Pérez, P. Pérez-Soriano, S. Llana Belloch, Á. G. Lucas-Cuevas, and D. Sánchez-Zuriaga, "Effects of treadmill running and fatigue on impact acceleration in distance running," *Sport. Biomech.*, vol. 13, no. 3, pp. 259–266, 2014, doi: 10.1080/14763141.2014.909527.

[12] E. C. Hardin and J. Hamill, "The influence of midsole cushioning on mechanical and hematological responses during a prolonged downhill run," *Res. Q. Exerc. Sport*, vol. 73, no. 2, pp. 125–133, 2002, doi: 10.1080/02701367.2002.10609001.

[13] T. Hoenig, D. Hamacher, K. M. Braumann, A. Zech, and K. Hollander, "Analysis of running stability during 5000 m running *," *Eur. J. Sport Sci.*, vol. 19, no. 4, pp. 413–421, 2019, doi:

10.1080/17461391.2018.1519040.

[14] A. G. Lucas-Cuevas, A. Camacho-García, R. Llinares, J. I. P. Quesada, S. Llana-Belloch, and P. Pérez-Soriano, "Influence of custom-made and prefabricated insoles before and after an intense run," *PLoS One*, vol. 12, no. 2, pp. 1–14, 2017, doi: 10.1371/journal.pone.0173179.

[15] R. S. McGinnis, S. M. Cain, S. P. Davidson, R. V. Vitali, N. C. Perkins, and S. G. McLean, "Quantifying the effects of load carriage and fatigue under load on sacral kinematics during countermovement vertical jump with IMU-based method," *Sport. Eng.*, vol. 19, no. 1, pp. 21–34, 2016, doi: 10.1007/s12283-015-0185-3.

[16] S. A. Meardon, J. Hamill, and T. R. Derrick, "Running injury and stride time variability over a prolonged run," *Gait Posture*, vol. 33, no. 1, pp. 36–40, Jan. 2011, doi: 10.1016/j.gaitpost.2010.09.020.

[17] J. A. Mercer, B. T. Bates, J. S. Dufek, and A. Hreljac, "Characteristics of shocks attenuation during fatigued running," *J. Sports Sci.*, vol. 21, no. 11, pp. 911–919, 2003, doi: 10.1080/0264041031000140383.

[18] F. Meyer, M. Falbriard, B. Mariani, K. Aminian, and G. P. Millet, "Continuous Analysis of Marathon Running Using Inertial Sensors: Hitting Two Walls?," *Int. J. Sports Med.*, vol. 42, no. 13, pp. 1182–1190, 2021, doi: 10.1055/a-1432-2336.

[19] J. Mizrahi, O. Verbitsky, and E. Isakov, "Shock accelerations and attenuation in downhill and level running." Accessed: Feb. 04, 2021. [Online]. Available: www.elsevier.com/locate/clinbiomech.

[20] J. Mizrahi, O. Verbitsky, and E. Isakov, "Fatigue-related loading imbalance on the shank in running: a possible factor in stress fractures," *Ann. Biomed. Eng.*, vol. 28, no. 4, pp. 463–469, 2000, doi: 10.1114/1.284.

[21] J. Mizrahi, O. Verbitsky, E. Isakov, and D. Daily, "Effect of fatigue on leg kinematics and impact acceleration in long distance running," *Hum. Mov. Sci.*, vol. 19, no. 2, pp. 139–151, 2000, doi: 10.1016/S0167-9457(00)00013-0.

[22] J. Mizrahi, O. Verbitsky, and E. Isakov, "Fatigue-induced changes in decline running," *Clin. Biomech.*, vol. 16, no. 3, pp. 207–212, 2001, doi: 10.1016/S0268-0033(00)00091-7.

[23] M. KA and M. BM, "Effect of fatigue on tibial impact accelerations and knee kinematics in drop jumps," *Med. Sci. Sports Exerc.*, vol. 38, no. 10, pp. 1836–1842, Oct. 2006, doi: 10.1249/01.MSS.0000229567.09661.20.

[24] C. Morio, V. Sevrez, P. Chavet, E. Berton, and C. Nicol, "Neuro-mechanical adjustments to shod versus barefoot treadmill runs in the acute and delayed stretch-shortening cycle recovery phases," *J. Sports Sci.*, vol. 34, no. 8, pp. 738–745, 2016, doi: 10.1080/02640414.2015.1069379.

[25] T. Provot, A. Najdem, L. Valdes-Tamayo, and X. Chiementin, "Impact of exhaustion level on tibial acceleration signature of amateur runners," *Comput. Methods Biomech. Biomed. Engin.*, vol. 23, no. sup1, pp. S241–S243, 2020, doi: 10.1080/10255842.2020.1815322.

[26] J. Reenalda, E. Maartens, L. Homan, and J. H. (Jaap. Buurke, "Continuous three dimensional analysis of running mechanics during a marathon by means of inertial magnetic measurement units to objectify changes in running mechanics," *J. Biomech.*, vol. 49, no. 14, pp. 3362–3367,

Oct. 2016, doi: 10.1016/j.jbiomech.2016.08.032.

[27] J. Reenalda, E. Maartens, J. H. Buurke, and A. H. Gruber, "Kinematics and shock attenuation during a prolonged run on the athletic track as measured with inertial magnetic measurement units," *Gait Posture*, vol. 68, pp. 155–160, Feb. 2019, doi: 10.1016/j.gaitpost.2018.11.020.

[28] M. Ruder, S. T. Jamison, A. Tenforde, F. Mulloy, and I. S. Davis, "Relationship of Foot Strike Pattern and Landing Impacts during a Marathon," *Med. Sci. Sports Exerc.*, vol. 51, no. 10, pp. 2073–2079, Oct. 2019, doi: 10.1249/MSS.0000000000002032.

[29] M. A. Sandrey, Y. J. Chang, and J. L. McCrory, "The effect of fatigue on leg muscle activation and tibial acceleration during a jumping task," *J. Sport Rehabil.*, vol. 29, no. 8, pp. 1093–1099, 2020, doi: 10.1123/JSR.2018-0495.

[30] K. H. Schütte, E. A. Maas, V. Exadaktylos, D. Berckmans, R. E. Venter, and B. Vanwanseele, "Wireless tri-axial trunk accelerometry detects deviations in dynamic center of mass motion due to running-induced fatigue," *PLoS One*, vol. 10, no. 10, pp. 1–12, 2015, doi: 10.1371/journal.pone.0141957.

[31] K. H. Schütte, S. Seerden, R. Venter, and B. Vanwanseele, "Influence of outdoor running fatigue and medial tibial stress syndrome on accelerometer-based loading and stability," *Gait Posture*, vol. 59, no. October 2017, pp. 222–228, 2018, doi: 10.1016/j.gaitpost.2017.10.021.

[32] C. Strohrmann, H. Harms, C. Kappeler-Setz, and G. Tröster, "Monitoring kinematic changes with fatigue in running using body-worn sensors," *IEEE Trans. Inf. Technol. Biomed.*, vol. 16, no. 5, pp. 983–990, 2012, doi: 10.1109/TITB.2012.2201950.

[33] O. Verbitsky, J. Mizrahi, A. Voloshin, J. Treiger, and E. Isakov, "Shock transmission and fatigue in human running," *J. Appl. Biomech.*, vol. 14, no. 3, pp. 300–311, 1998, doi: 10.1123/jab.14.3.300.

[34] W. Luo *et al.*, "Guidelines for Developing and Reporting Machine Learning Predictive Models in Biomedical Research: A Multidisciplinary View," *J Med Internet Res 2016;18(12)e323 https//www.jmir.org/2016/12/e323*, vol. 18, no. 12, p. e5870, Dec. 2016, doi: 10.2196/JMIR.5870.

[35] S. Ameli, F. Naghdy, D. Stirling, G. Naghdy, and M. Aghmesheh, "Quantitative and non-invasive measurement of exercise-induced fatigue," *Proc. Inst. Mech. Eng. Part P J. Sport. Eng. Technol.*, vol. 233, no. 1, pp. 34–45, 2019, doi: 10.1177/1754337118775548.

[36] D. Arias-Torres, J. A. Hernández-Nolasco, M. A. Wister, and P. Pancardo, "Detection of fatigue on gait using accelerometer data and supervised machine learning," *Int. J. Grid Util. Comput.*, vol. 11, no. 4, pp. 474–485, 2020, doi: 10.1504/IJGUC.2020.108475.

[37] S. Hajifar, H. Sun, F. M. Megahed, L. A. Jones-Farmer, E. Rashedi, and L. A. Cavuoto, "A forecasting framework for predicting perceived fatigue: Using time series methods to forecast ratings of perceived exertion with features from wearable sensors," *Appl. Ergon.*, vol. 90, p. 103262, Jan. 2021, doi: 10.1016/J.APERGO.2020.103262.

[38] Y. Jiang, V. Hernandez, G. Venture, D. Kulić, and B. K. Chen, "A data-driven approach to predict fatigue in exercise based on motion data from wearable sensors or force plate," *Sensors*, vol. 21, no. 4, pp. 1–16, 2021, doi: 10.3390/s21041499.

[39] S. Karvekar, M. Abdollahi, and E. Rashedi, "Smartphone-based human fatigue level detection

using machine learning approaches," *https://doi.org/10.1080/00140139.2020.1858185*, vol. 64, no. 5, pp. 600–612, 2021, doi: 10.1080/00140139.2020.1858185.

[40] T. Provot, X. Chiementin, F. Bolaers, and M. Munera, "A time to exhaustion model during prolonged running based on wearable accelerometers," *Sport. Biomech.*, vol. 20, no. 3, pp. 330–343, 2021, doi: 10.1080/14763141.2018.1549682.

[41] J. Zhang, T. E. Lockhart, and R. Soangra, "Classifying lower extremity muscle fatigue during walking using machine learning and inertial sensors," *Ann. Biomed. Eng.*, vol. 42, no. 3, pp. 600–612, 2014, doi: 10.1007/s10439-013-0917-0.
